# Long‐Term Depth Records of Satellite‐Tagged Northern Bottlenose Whales Reveal Extraordinary Dive Capabilities

**DOI:** 10.1002/ece3.71862

**Published:** 2025-07-30

**Authors:** Barbara K. Neubarth, Patrick J. O. Miller, Rune Roland, Lars Kleivane, Paul J. Wensveen

**Affiliations:** ^1^ Faculty of Life and Environmental Sciences, School of Engineering and Natural Sciences University of Iceland Reykjavík Iceland; ^2^ Coastal and Marine Management University Centre of the Westfjords Ísafjörður Iceland; ^3^ Sea Mammal Research Unit Scottish Oceans Institute, School of Biology, University of St Andrews St Andrews UK; ^4^ University of Oslo Marine Biological Station Drøbak Norway; ^5^ LKARTS‐Norway Skutvik Norway; ^6^ Westman Islands Research Centre Institute of Research Centres, University of Iceland Vestmannaeyjar Iceland

**Keywords:** beaked whale, biotelemetry tags, cetacean, deep‐diving, dive behavior

## Abstract

Studying the baseline behavior of deep‐diving mammals can substantially improve our understanding of these species' ecology and provide important benchmarks to evaluate effects of changes in climate and anthropogenic activities. Despite being the most abundant beaked whale in the Arctic and subarctic, information on the behavior of northern bottlenose whales ( *Hyperoodon ampullatus* ) is limited. This study used records from 13 satellite tags deployed off Jan Mayen in June–July 2014–2016 to provide an extensive description of the dive behavior of *Hyperoodon* for the Nordic Seas. A total of 8372 dives, collected over 224 days (or 5376 h), were analyzed. The whales performed extreme dives of up to 2288 m deep and 98 min long—deeper and longer than previously reported for behavior in presumed undisturbed contexts. Individuals spent on average 18% of the time at depths shallower than 40 m, and 22%, 47%, and 12% in epi‐, meso‐, and bathypelagic dives, respectively. Epipelagic dives averaged 123 m (s.d.: 46 m) in depth and 11 min (5 min) in duration. Mesopelagic dives averaged 441 m (217 m) and 24 min (11 min) and were performed at a mean rate of 1.46 h^−1^. Bathypelagic dives averaged 1487 m (366 m) and 55 min (13 min) and were performed at a mean rate of 0.23 h^−1^. The distribution of dive depths was less bimodal than typically reported for other beaked whales, and all dive profiles contained periods of continuous, consecutive deep dives. Benthic diving occurred at meso‐ and especially bathypelagic depths and was individual specific, varying from 8% to 51% of the animal's bathypelagic dives. Overall, our findings demonstrate that northern bottlenose whales have extraordinary capabilities to dive, and presumably feed, throughout the water column including at the sea floor. High rates of deep dives highlight the importance of the Iceland and Norwegian Seas to this population of deep‐sea predators.

## Graphical abstract

This study used records from 13 satellite tags deployed off Jan Mayen to provide an extensive description of the dive behavior of northern bottlenose whales in the Nordic Seas. Our findings demonstrate that this species has extraordinary capabilities to dive, and presumably feed, throughout the water column including at the sea floor. High rates of deep dives highlight the importance of the Iceland and Norwegian Seas habitat to this population of deep‐sea predators.

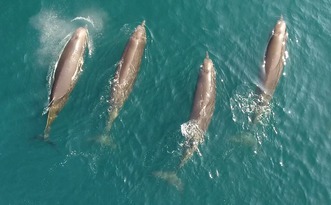

## 1 Introduction

Deep‐diving cetaceans in general are thought to play important roles in ocean nutrient recycling and maintaining biodiversity; however, their fully marine lifestyle and preference for deep, offshore waters make these species difficult to study. Biologging and biotelemetry of dive data on these deep‐sea predators have offered unique insights into their foraging (Watwood et al. 2006; Shaff and Baird 2021), migratory (Pitman et al. 2020) and social behavior (Cioffi et al. 2021), physiology and habitat use (Hooker et al. 2009; Joyce et al. 2017), and predator–prey dynamics (Southall et al. 2019; Visser et al. 2021). Long‐term tags for deep‐diving cetaceans, only recently available for medium‐sized odontocetes, can help identify areas of special conservation concern and show how their natural behavior may vary between seasons (e.g., Storrie et al. 2022).

Beaked whales (family *Ziphiidae*) are cryptic, medium‐sized odontocetes known for their extreme deep‐diving and their inconspicuous behavior at the surface (Hooker et al. 2019). Their long and deep dives are typically single dives that are followed by several shorter and shallower dives that have, in part, a respiratory function (Baird et al. 2008; Minamikawa et al. 2007). Deep dives of beaked whales are associated with foraging at meso‐/bathypelagic depths, where the whales hunt for small fish and deep‐water squid at very high rates (Madsen et al. 2014). For example, Blainville's beaked whales ( *Mesoplodon densirostris* ) off El Hierro in the Canary Islands were found to forage near steep bathymetric slopes on stable and abundant prey resources near the deep scattering and bottom boundary layers (Arranz et al. 2011). Moreover, beaked whales are known to undertake conspicuously long and deep dives in response to perceived threats such as killer whale predators or human‐made disturbance (Miller et al. 2015).

**FIGURE 1:**
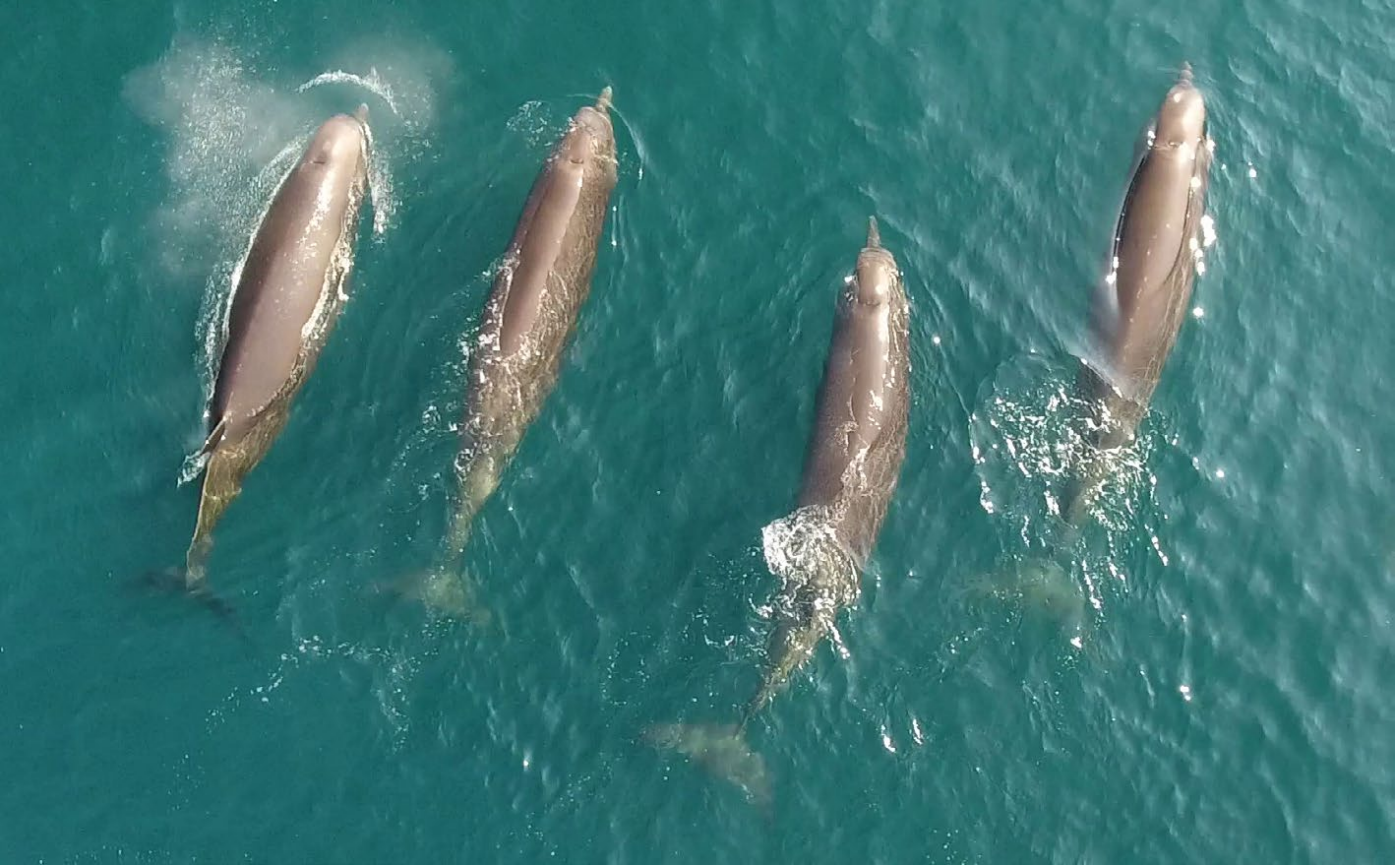
Aerial photograph of a group of northern bottlenose whales ( *Hyperoodon ampullatus* ) swimming at the surface. Photographer: Patrick Kagerer.

Northern bottlenose whales ( *Hyperoodon ampullatus* ; Figure 1) are found in most of the North Atlantic, ranging from the Azores, Portugal, in the South to Arctic Svalbard in the North (Silva et al. 2014; Whitehead and Hooker 2012), and are the only beaked whale species regularly seen in subarctic and Arctic waters (Gowans 2009; Madsen et al. 2014). They are most often sighted in waters deeper than 500 m and close to subsea canyons and gorges, and near continental slopes (Feyrer, Stanistreet, Gomez, et al. 2024; Pike et al. 2019; Wimmer and Whitehead 2004). The species' diet is thought to consist predominantly of deep‐water squid, which makes these whales specialized niche feeders (Fernández et al. 2014; Hooker, Baird, et al. 2001; Hooker, Iverson, et al. 2001). Their favored habitat seems to be correlated with this choice of prey (Bjørke 2001).

Dive behavior of northern bottlenose whales was first studied using suction‐cup attached time‐depth recorders in 1996–1998 in the Northwest Atlantic (Hooker and Baird 1999). Two individuals dove to depths greater than 800 m approximately every 80 min, with dives lasting up to 70 min (Hooker and Baird 1999). In 2013–2016, 15 multisensor DTAGs were deployed on northern bottlenose whales off Jan Mayen in the Northeast Atlantic for studies on behavior and body condition (Miller et al. 2016; Siegal et al. 2022; Haas et al. 2025). Extraordinary dives were observed, with the deepest dive reaching a depth of 2339 m (Miller et al. 2015) and the longest dive lasting 2 h and 10 min (Wensveen et al. 2019). However, the whales conducted these extreme dives in response to controlled naval sonar exposures (Miller et al. 2015; Wensveen et al. 2019) and thus such dive parameters may be outside of their natural repertoire.

The objective of this study was to provide a comprehensive description of the dive behavior of northern bottlenose whales in the Nordic Seas—a key area for this population (Benjaminsen and Christensen 1979; Pike et al. 2019)—using dive summary information from satellite tags. So far, the published knowledge of this species' dive behavior is mainly derived from short‐term, high‐resolution tag data (e.g., Hooker and Baird 1999) allowing brief (hours to days), detailed insights into the whales' life below the ocean surface. Depth‐transmitting satellite tags are an important complementary technique, providing courser data on diving and surface behavior but over much longer time intervals (days to months), appropriate for this study.

## 2 Materials and Methods

### 2.1 Data Collection and Tag Programming

**FIGURE 2:**
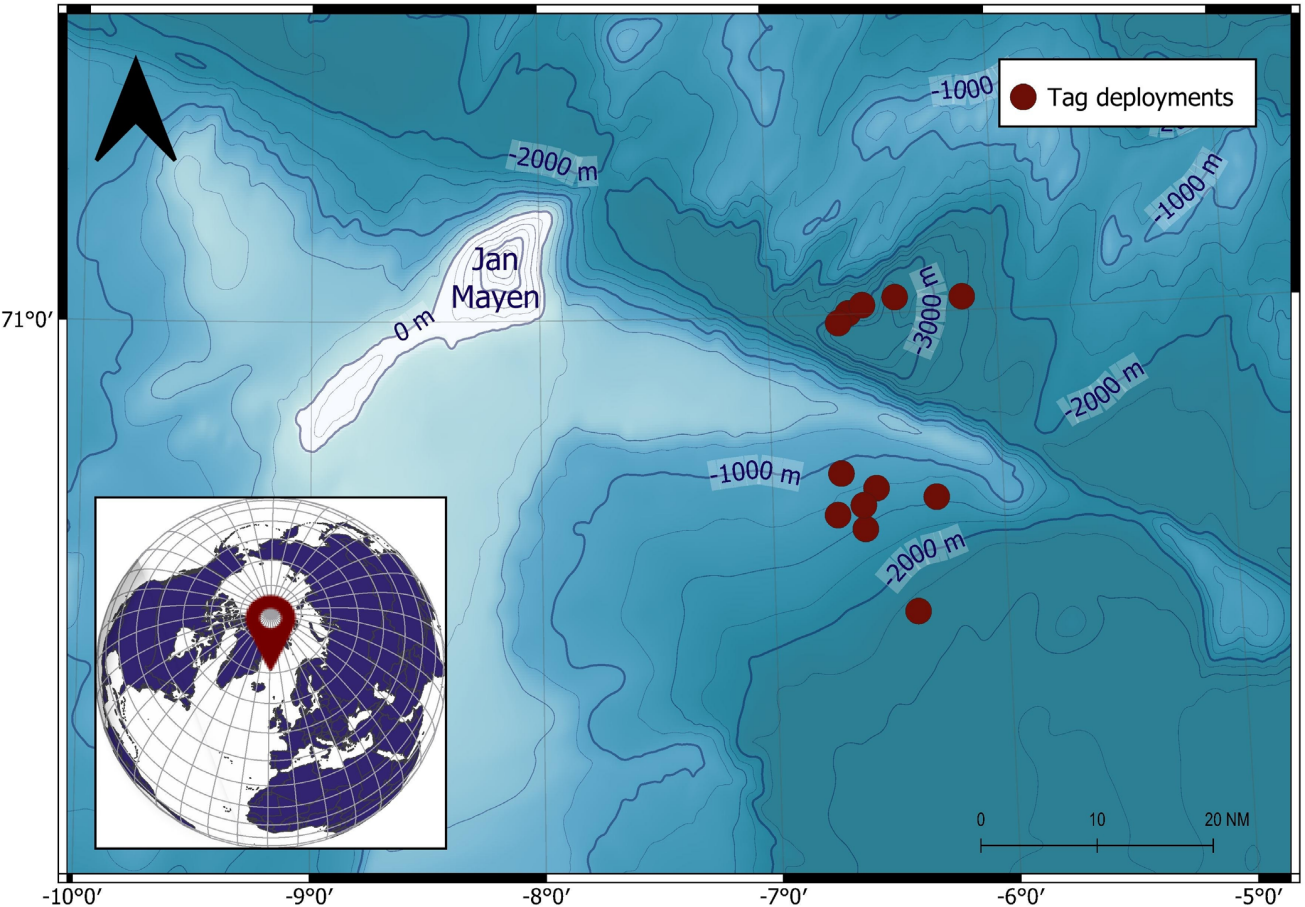
Map of the waters near Jan Mayen, showing the locations of the satellite tag deployments on northern bottlenose whales that were analyzed in this study. Bathymetry data for the map were obtained from the ETOPO 2022 global relief model (NOAA 2022).

We tagged 13 individuals with SPLASH10 satellite tags (Wildlife Computers, Redmond, WA) off Jan Mayen (71.0° N 8.5° W; Norway) from June 2014 to 2016 (Table 1; Figure 2) using an air‐gun—the ARTS (Kleivane et al. 2022) or JM Special 25 (DanInject, Kolding, Denmark). We deployed the regular depth (292A; *n* = 3 individuals) and extended depth (292B; *n* = 10 individuals) versions of the tag (Table S1), with depth ratings of 2000 and 3000 m, respectively (Shearer et al. 2019). The tag was attached to the whale by two 68‐mm titanium darts with backward‐facing petals. We aimed to attach the tag to the dorsal fin to achieve maximum deployment durations (Dietz et al. 2020) and to maximize data throughput (Quick et al. 2019). The tags and darts were sterilized using a 70% ethanol solution before deployment. Tagging procedures followed best‐practice guidelines and included an evaluation of behavioral reactions (Hooker, Baird, et al. 2001; Hooker, Iverson, et al. 2001) and photo and video documentation during the event (Andrews et al. 2019). We only tagged adults, subadults, or juveniles. The research protocols were permitted by the Norwegian Animal Research Authority (permit nos. S2011/38782 and 2015/23222) and approved by the University of St Andrews Animal Welfare and Ethics Committee.

**TABLE 1:** Overview of the depth‐transmitting satellite tag deployments on northern bottlenose whales in the Nordic Seas, including tag‐on time and location, tagging method, and if the tag went into the blubber below the dorsal fin or attached to the dorsal fin. Deployment duration is the time between tag‐on and the end of the dive record. Also provided is the individual's number in the photo‐identification catalog, its sex‐age class (FJ, Female/juvenile; MM, Mature male; Feyrer et al. 2021), and its reaction to the tag attachment on a 4‐point scale (Hooker, Baird, et al. 2001; Hooker, Iverson, et al. 2001).

Tag ID	Date/time (UTC)	Tag‐on location		Tagging method	Tag placement	Reaction to tagging	Deployment duration	Sex/age	Individual ID
		Latitude (°)	Longitude (°)			(0–3)^a^			
134664	22/06/2014 19:45	70.9674	−6.0894	ARTS	Blubber	1	1d 15 h	—	038
134666	23/06/2014 09:15	70.825	−5.9961	ARTS	Blubber	1	0 h	—	309
134663	24/06/2014 23:28	70.6978	−5.6848	ARTS	Blubber	1	5 h	FJ	5114
134670	22/06/2015 14:45	70.9859	−6.6736	Dan Inject	Dorsal fin	1	41d 21h^b^	FJ	—
134669	22/06/2015 15:59	70.9565	−6.7595	Dan Inject	Base of dorsal fin	1	8d 15 h	—	126
134668	23/06/2015 20:42	70.9859	−6.6736	Dan Inject	Dorsal fin	1	26d 19 h	MM	235
161587	15/06/2016 22:04	70.7449	−6.5316	Dan Inject	Dorsal fin	1	33d 19 h	FJ	—
161588	15/06/2016 22:38	70.7354	−6.5663	ARTS	Dorsal fin	1	33d 2 h	FJ	366
161590	16/06/2016 04:30	70.7618	−6.5392	Dan Inject	Dorsal fin	1	31d 20 h	FJ	—
161592	18/06/2016 08:13	70.7407	−6.494	Dan Inject	Blubber	1	3d 11 h	FJ	5280
161593	18/06/2016 08:15	70.7407	−6.4934	Dan Inject	Dorsal fin	1	37d 17h^b^	FJ	—
161591	18/06/2016 10:30	70.7588	−6.5075	ARTS	Blubber	1	4d 0 h	FJ	368
134667	21/06/2016 09:05	71.0595	−6.8208	Dan Inject	Dorsal fin	1	0d 20 h	FJ	384

*Note:* Gray shading indicates deployments of 4 days or less.

^a^ Reactions of the whales to the tagging process were scored from 0 to 3. 0/No reaction = no detectable change; 1/Low‐level reaction = short‐term mild change, for example, flinch, fast dive; 2/Moderate = short‐term forceful change, for example, breach; 3/Strong = succession of forceful activities.

^b^ Durations for period in the Nordic Seas (excluding the North Atlantic): 12 days and 12 h (ID 134670) or 18 days and 5 h (ID 161593).

Photographs of the tagged whale's dorsal fin and the shape of its forehead (or, “melon”) were taken during the encounter. The best photograph of the dorsal fin was compared to a photo‐identification catalog for the Iceland–Jan Mayen–Faroes region using methods described in Neubarth and Wensveen (2021). To determine the sex‐age class of the tagged whale, the shape of the melon was classified following Feyrer et al. (2021).

The tags were programmed to collect and transmit continuous records of dive summary information using the Behavior Log function from Wildlife Computers. Depending on the deployment, the tag's onboard processing used either the wet/dry sensor readings or a depth threshold of 5 m to define the start and end of a dive (see Table S1, also for other tag settings). The dive summary variables were the maximum dive depth, dive duration, dive shape (U, V, or square), duration of the surface period between dives, and UTC start time and end time of the dive or surface period (Wildlife Computers 2020). For maximum dive depth, the tag recorded a lower and upper limit, and the linear average of these was used in the analysis. A dive had to be at least 40 m deep to be included in the dive analysis (*sensu* Hooker and Baird 1999) and shallower dives were considered part of surface periods. On every 6th day (2014–2015) or 7th day (2016) of the deployment, the tag sampled depth data at 2.5‐min resolution, which were used to groundtruth the dive summary information (Table S1). Data were transmitted by the tag to the ARGOS satellite system, and subsequently downloaded and processed via the DAP program (Wildlife Computers 2021).

### 2.2 Data Processing

Data processing and statistical analyses were executed in R (v4.4.0) using the interface RStudio. Tag records were checked for biologically implausible values of vertical velocity or technical failure following Cioffi et al. (2023) and Shearer et al. (2019). All dives were considered plausible; only three (shallow) dives had an absolute mean vertical velocity above 2.0 m s^−1^ and none were above 2.5 m s^−1^. Dive summary data were considered missing (a “data gap”) if the interval between the end of a dive or surface period and the start of the subsequent one was longer than 60 s. Because only natural, undisturbed behavior was of interest, we conservatively excluded data collected (1) during the first 24 h after tag‐on, to account for potential tagging effects, and (2) during and directly after sonar exposure experiments. A period of 24 h following exposure was excluded for 2015, whereas 48 h following exposure was excluded for 2016. In 2015, the satellite‐tagged whales were exposed to low sonar received levels of 62–82 dB re 1 μPa and behavioral responses were not apparent (Wensveen et al. 2019). In 2016, in contrast, all tagged whales exhibited behavioral responses at sonar received levels of 120–126 dB re 1 μPa and their horizontal movement and dive behavior was considered back to baseline levels after 8 to 16 h (von Benda‐Beckmann et al. 2019; Wensveen et al. 2019).

Low‐resolution dive profiles were created from the dive summary information in the Behavior Logs using average bottom times based on the tag's dive shape definitions (Wildlife Computers 2020). These dive profiles consisted of symmetrical dives that were either square‐, U‐, or V‐shaped, with bottom times of 75%, 35%, and 10% of the total dive time, respectively. They were overlaid onto the 2.5‐min resolution time‐depth profiles and visually inspected for data gaps and differences in dive depth, duration, and shape. The dive durations were not always consistent between the two data series, and some dives had noticeably different durations (Figures S5–S10). The cause of this issue was discovered to be an erroneous setting used in 2014–2015. Although the wet/dry and pressure sensors were sampled every 1 s, the sampling interval for histogram data, which also applies to the Behavior Log, was set to 10 s in 2014–2015 and 1 s in 2016 (Table S1). This setting caused the tags in 2014–2015 to downsample the wet/dry sensor readings to 0.1 Hz and led to undetected breaths during surface periods, which biased measurements of surface and dive duration. The dive profiles from 2016 (Figures S11–S16) did not contain such irregularities. Therefore, the surface and dive durations from 2014 to 2015 were excluded from further analysis.

To be able to link spatial variables to the dive summaries, we predicted the whales' locations (Figure S3) by fitting continuous‐time correlated random walk models (Johnson et al. 2008) to the raw ARGOS tracks (details in Wensveen et al. 2019). The bathymetric depth halfway between the start and end of each dive was obtained from the 15‐s resolution SRTM15+ global bathymetry data set, which was accessed via the R package *rerddap* (v1.1.0, Chamberlain et al. 2024). Two of the tagged whales (IDs 134670 and 161593) crossed the Iceland–Faroe Ridge, which rises to depths of ~500 m or less along the middle of the ridge (Hansen and Østerhus 2000). The Iceland–Faroe Ridge is a section of the Greenland–Scotland Ridge, an important barrier between the colder, fresher water in the Nordic Seas and the warmer, more saline water in the central North Atlantic (Pampoulie et al. 2024). After leaving the Nordic Seas, these two whales appeared to switch to a more directed migratory behavior, comparable to that of northern bottlenose whales that were tagged in the Canadian Arctic (Lefort et al. 2025). Internal and external factors can influence behavior, and the objective was to describe the species' dive behavior for the Nordic Seas region. The species is most often encountered in waters deeper than 800 m (Ramirez‐Martinez et al. 2024; Whitehead and Hooker 2012); therefore, we used the 800‐m depth contour at the north side of the Iceland–Faroe Ridge to exclude dives (10% of the total) and surface periods that occurred above or south of the Ridge (Figure S3).

### 2.3 Statistical Analysis

To evaluate within which oceanographic zones the whales dived, each dive was categorized based on its maximum depth as epipelagic (0–200 m), mesopelagic (200–1000 m), or bathypelagic (1000–4000 m). The first foraging buzzes recorded using DTAGs in Jan Mayen typically occurred at 100–300 m depth, and most undisturbed dives deeper than 200 m included regular echolocation and buzz sounds from the tagged whales (Miller et al. 2015; Haas et al. 2025). Therefore, the a priori categorization of dives was partially informed by DTAG data and considered biologically relevant for northern bottlenose whales in the study area. To look further into the potential function of dives, we compared dive shape occurrence (square, U, V) within each dive category. Additionally, we also considered whether dives were benthic (defined as reaching within 100 m or beyond the charted ocean floor depth according to the SRTM15+ data). We expected the estimates of proximity to the ocean floor to be sensitive to error due to uncertainties in bathymetry and whale locations, especially in areas with steep bathymetric gradients, but their errors to be comparable across tag deployments. Mean dive rate was calculated as the total number of dives divided by the sum of all dive and surface durations. We defined inter‐deep‐dive interval (IDDI) as the time between adjacent meso–/bathypelagic dives (i.e., dives deeper than 200 m). The IDDI is a common metric used to describe beaked whale dive behavior, as deep dives are typically associated with foraging (Tyack et al. 2006). Here, we refer to all meso–/bathypelagic dives as deep dives even though a depth threshold of 200 m is relatively shallow for beaked whales. Because reliable dive and surface durations were only available for 2016, descriptive statistics of these variables and of the dive rates, shapes, and intervals were calculated only for the 2016 subset. Spearman's rank correlations were used due to nonnormality of the variables. Dependent correlations were statistically compared using Fisher's *z* tests implemented in the R package *diffcor* (v0.8.3, Blötner 2024).

## 3 Results

### 3.1 Overview of the Data Set

Thirteen satellite tags were successfully deployed in 2014–2016, with a range of deployment durations from 0 h to 41.9 days (Table 1) and a total duration of 224 days (or 5376 h). One tagged whale was identified as a mature male (ID 134668), nine were identified as female/juvenile, and three were of unknown sex‐age class (Table 1). We only observed low‐level reactions to the tagging where the whale modified its behavior slightly, for example, dived rapidly. The tag's location on the body of the whale greatly affected the deployment duration, averaging 2 days (range: 0–4 days; *n* = 5 tags) for attachment below the dorsal fin and 27 days (range: 1–42 days; *n* = 8 tags) for attachment to the dorsal fin (Table 1). The latter increased to 31 days if an older tag deployed with a relatively low battery voltage (ID 134667) was excluded. Three tag records either only contained dive data in the first 24 h after tagging (IDs 134663 and 134667) or not at all (ID 134666) so were excluded from further analyses. None of the remaining 10 dive records (*n* = 4 from 2014 to 2015; *n* = 6 from 2016) were continuous, and the average data gap lasted 5.2 h (s.d.: 10.7 h; max: 3.6 days). After excluding data that could be affected by sonar and tagging responses or migration, the 10 tag records included a total of 7531 dives that were deeper than 40 m, of which 5768 dives (76%) were recorded in 2016 (Table 2). Categorizing dives by oceanographic zone resulted in 3629 (48%) epipelagic dives (40–200 m deep), 3377 (45%) mesopelagic dives (200–1000 m deep) and 525 (7%) bathypelagic dives (> 1000 m deep) (Table 2).

**TABLE 2:** The numbers and percentages of epipelagic (40–200 m), mesopelagic (200–1000 m), and bathypelagic (> 1000 m) dives of northern bottlenose whales in the Nordic Seas that were analyzed in this study. A dive was defined by a minimum depth of 40 m (Hooker and Baird 1999). The percentage of dives that were considered benthic (within 100 m from the ocean floor) and summary statistics for maximum dive depth are also provided. The deployments are listed in chronological order.

Year	Tag ID	No. dives			Benthic dives			Dive depth (m)	
		*epi*	*meso*	*bathy*	*epi*	*meso*	*bathy*	Mean ± SD	Maximum
2014	134664	7 (54%)	6 (46%)	—	—	—	—	269 ± 212	840
2015	134670	369 (71%)	106 (20%)	43 (8%)	1%	12%	51%	320 ± 428	2160
2015	134669	65 (53%)	40 (33%)	17 (14%)	—	8%	35%	411 ± 468	1904
2015	134668	496 (45%)	465 (42%)	149 (13%)	—	1%	17%	441 ± 457	1848^a^
2016	161587	836 (51%)	795 (48%)	22 (1%)	—	1%	23%	317 ± 261	1520
2016	161588	583 (35%)	931 (56%)	157 (9%)	—	1%	8%	398 ± 456	2288
2016	161590	625 (45%)	693 (49%)	85 (6%)	—	3%	34%	367 ± 355	2096
2016	161592	17 (36%)	25 (53%)	5 (11%)	—	—	20%	349 ± 387	1936
2016	161593	584 (65%)	269 (30%)	44 (5%)	—	—	45%	289 ± 344	2160
2016	161591	47 (48%)	47 (48%)	3 (3%)	—	—	—	302 ± 288	1936
All tags		3629 (48%)	3377 (45%)	525 (7%)	0%	2%	23%	361 ± 387	2288

*Note:* Gray shading indicates deployments of 4 days or less.

^a^ ID 134668 was a tag without the extended depth range and was only capable of measuring depth up to 1848 m.

### 3.2 Description of Northern Bottlenose Whale Dive Behavior

**FIGURE 3:**
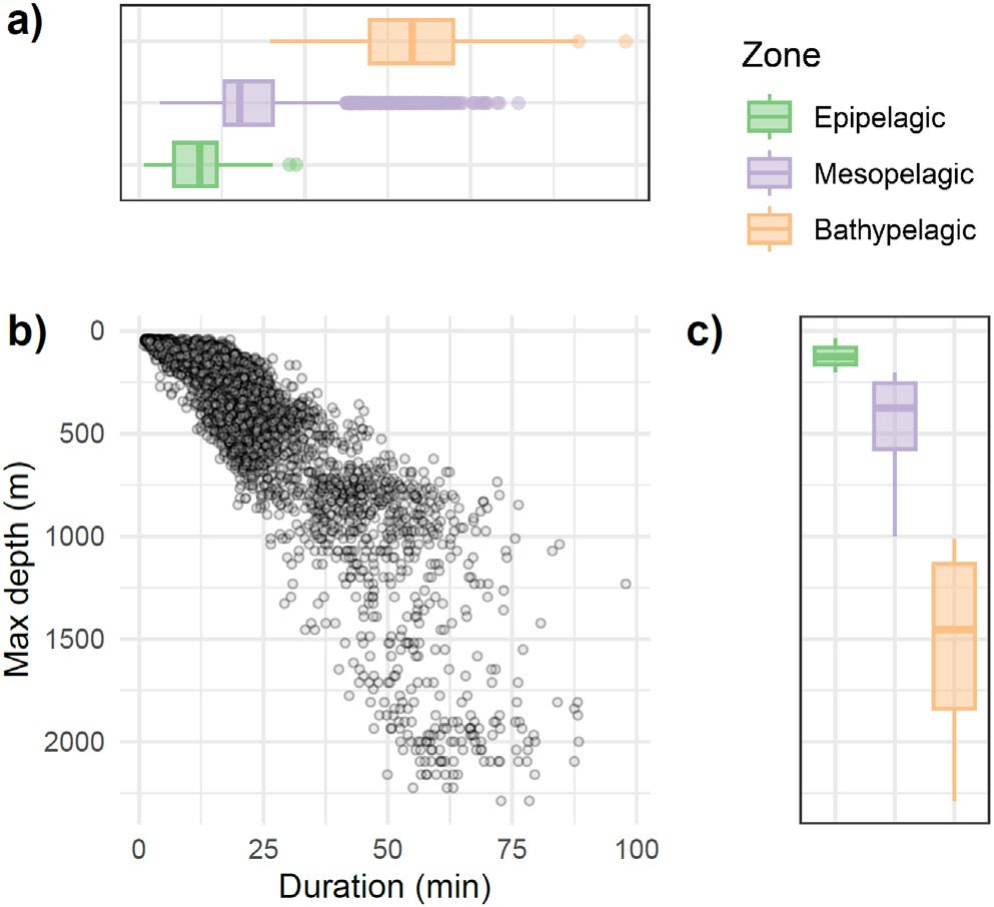
(a) Boxplots of dive durations measured by satellite tags on northern bottlenose whales in 2016 per oceanographic zone (b) scatterplot of maximum depth and duration of the dives measured in 2016 (c) boxplots of maximum dive depths per oceanographic zones throughout all deployments (2014, 2015, 2016). Note the depth axis is positive downwards.

Maximum dive depth averaged 361 m (s.d.: 387 m), with individual averages ranging between 269 and 441 m (Table 2), and was strongly correlated with dive duration (*ρ* = +0.86; 2016 only) (Figure 3b). Epipelagic dives averaged 11 min (s.d.: 5 min) in duration and were performed at a mean rate of 1.15 per hour (Table 3; 2016 only). Mesopelagic dives lasted on average 24 min (s.d.: 11 min) and were performed at a similar mean rate as epipelagic dives of 1.18 per hour (2016 only; Table 3). Bathypelagic dives averaged 55 min (s.d.: 13 min) in duration and occurred at a much lower rate of 0.14 per hour (2016 only; Table 3). Surface periods (*n* = 6264; 2016 only) were generally much shorter than dives, with an average surface duration of 4.1 min (s.d.: 3.1 min; max: 55.7 min) (Table 3). On average, northern bottlenose whales spent 18% of their time at depths shallower than 40 m and 22%, 47%, and 12% of their time in epi‐, meso‐, and bathypelagic dives, respectively (2016 only). The most observed dive shape across all whales in 2016 was the U‐shape (68%), followed by the square‐shape (28%) and V‐shape (4%). The dive shape distribution across dive categories suggested that bottom time percentage decreased when dives were deeper; epi‐, meso‐, and bathypelagic dives were square‐shaped in 36%, 22%, and 2% of the dives, U‐shaped in 61%, 73%, and 84% of the dives, and V‐shaped in 3%, 5%, and 14% of the dives, respectively (2016 only).

**TABLE 3:** Descriptive statistics of the variables that were only calculated for tags deployed in 2016: Surface duration, dive duration, dive rate, and the interval between meso‐/bathypelagic dives. A dive was defined by a depth threshold of 40 m (Hooker and Baird 1999). Surface duration: Time between the end of a dive and the start of the next dive; dive rate: Mean number of dives per hour; IDDI, Inter‐deep dive interval. The mean, standard deviation, and range are provided for all variables except dive rate.

Tag ID	Surface duration (min)	Dive duration (min)			Dive rate (h^−1^)			IDDI (min)
		*epi*	*meso*	*bathy*	*epi*	*meso*	*bathy*	
161587	4.2 ± 2.6 [0–33.5]	12 ± 6 [1–30]	28 ± 13 [4–72]	53 ± 10 [38–84]	1.23	1.17	0.03	20 ± 27 [0–338]
161588	3.8 ± 2.4 [0.1–32.4]	11 ± 5 [1–32]	20 ± 8 [7–60]	54 ± 12 [26–88]	0.87	1.40	0.24	12 ± 19 [1–173]
161590	4.7 ± 4.3 [0–55.7]	11 ± 6 [1–26]	24 ± 10 [6–69]	57 ± 15 [29–88]	1.06	1.18	0.14	20 ± 45 [1–990]
161592	2.9 ± 1.1 [1.5–7.1]	16 ± 5 [4–24]	23 ± 6 [15–44]	59 ± 10 [51–73]	0.80	1.17	0.23	35 ± 64 [2–274]
161593	3.5 ± 2.3 [0–26]	12 ± 5 [1–26]	26 ± 12 [8–76]	60 ± 10 [44–98]	1.77	0.81	0.13	33 ± 45 [2–265]
161591	3.3 ± 1.7 [0.3–9.3]	10 ± 5 [2–19]	28 ± 15 [12–70]	63 ± 7 [54–67]	1.21	1.21	0.08	15 ± 25 [2–166]
All tags	4.1 ± 3.1 [0–55.7]	11 ± 5 [1–32]	24 ± 11 [4–76]	55 ± 13 [26–98]	1.15	1.18	0.14	18 ± 33 [0–990]

*Note:* Gray shading indicates deployments of 4 days or less.

**FIGURE 4:**
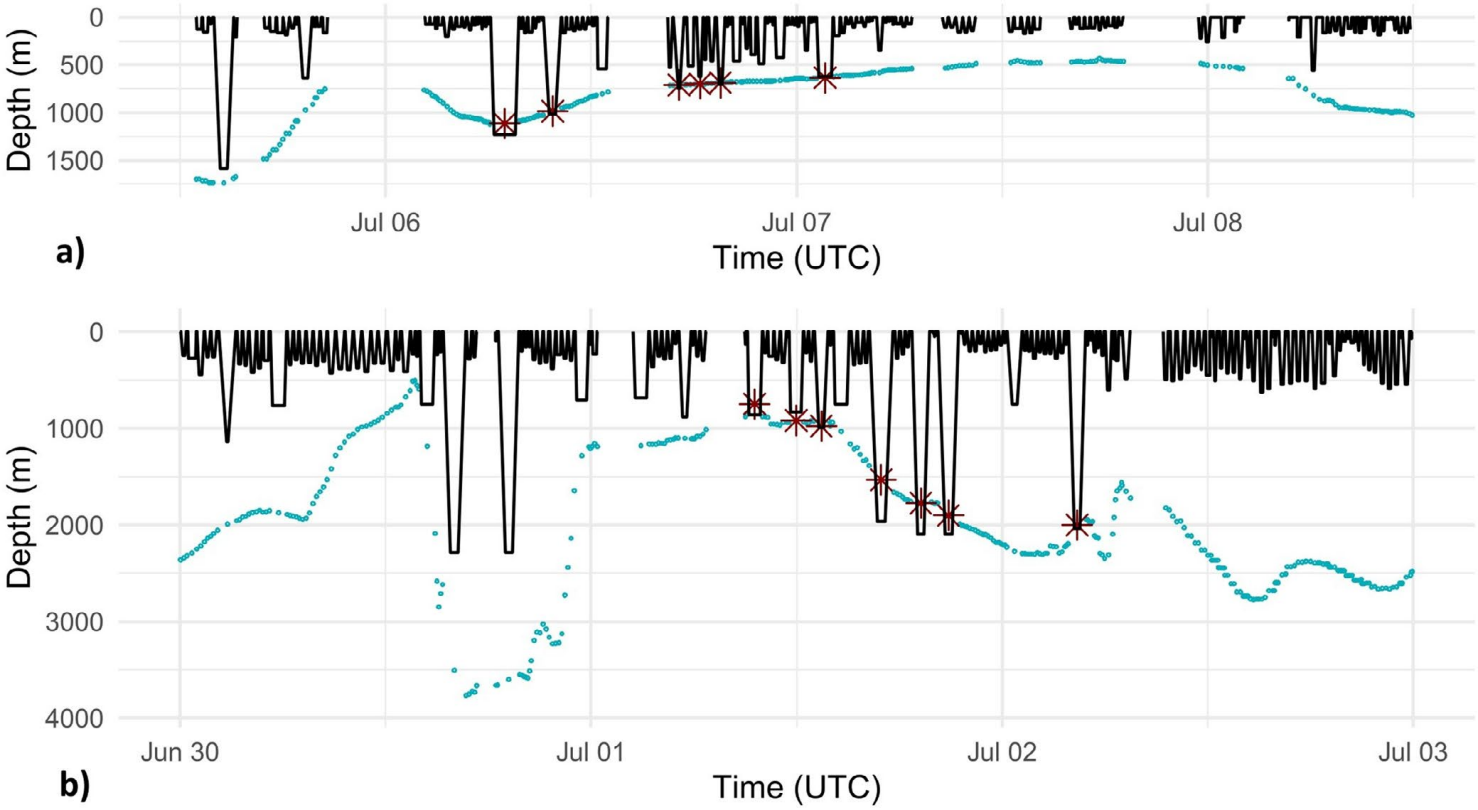
Dive profiles (black lines) reconstructed from dive summary information, containing the (a) longest dive (98 min; ID 161593) and (b) two deepest dives (both to 2288 m; ID 161588) in the data set. Turquoise dots: Seafloor depth; red asterisks: Dives considered benthic (< 100 from the charted seafloor depth).

Epi‐ and mesopelagic dives were commonly followed by dives of the same category (epipelagic: 56%; mesopelagic: 58%), whereas bathypelagic dives were rarely succeeded by another bathypelagic dive (6%). Indeed, the dive profile plots included many bouts of consecutive mesopelagic dives (Figure 4b and Figures S5–S16). The IDDI, that is, the time between two consecutive deep (i.e., meso−/bathypelagic) dives averaged 18 min and was highly variable (s.d.: 33 min), ranging from 0.0 to 16.5 h (Table 3). IDDI was positively correlated with the duration of the deep dive that came before (*ρ* = +0.30) and after (*ρ* = +0.47) (2016 only), suggesting that the northern bottlenose whales were taking longer to recover from prolonged anaerobic dives or were anticipating them by increasing oxygen intake. However, contrary to expectation, for the top 5% of dive durations (*sensu* Quick et al. 2020) of those deep dives (i.e., those longer than 58.7 min) the correlations were weaker and not significantly different (Figure S1).

Consistent differences in dive behavior across individuals were observed (Figure S2). One whale, a smaller individual (ID 161587), had a maximum recorded dive depth that was more than 300 m shallower than those of the other whales with tag deployments longer than 4 days (Table 2). Only 1% of this smaller whale's dives were categorized as bathypelagic (Table 2). In contrast, 14% of the dives of another whale (ID 134669) reached the bathypelagic zone, and this was 7% on average across all tag deployments. Epipelagic dives accounted for 35% to 71% (mean: 48%) of an individual's dives, depending on the deployment (Table 2). Mesopelagic dives ranged from 20% to 56% (mean: 45%) of an individual's dives, depending on the deployment (Table 2). The dive behavior of the only confirmed mature male (ID 134668) varied somewhat from that of the seven whales identified as mature female or juvenile. Despite the mature male carrying a tag without the extended depth range, his mean dive depth of 441 m was the deepest of all whales (Table 2). Additionally, the male was also the only individual that conducted two or more consecutive bathypelagic dives, suggesting an increased ability to repeatedly dive deep (Figure S8).

Benthic dives reached a depth of 1188 m (s.d.: 471 m) on average. After excluding all dives considered benthic, the maximum depth of the remaining deep dives was still correlated with the seafloor depth (*ρ* = +0.43). Individual whales exhibited varying degrees of benthic diving behavior, with the percentage of their bathypelagic dives that were also benthic varying between 8% and 51% (Table 2).

### 3.3 Longest and Deepest Dives Observed

The longest duration dive in the data set was conducted by an individual (ID 161593) that was classified as a female/juvenile. The dive started on 06 June 2016 at 06:08 UTC and had a duration of 98 min (Figure 4a). It was a benthic dive with a long bottom time (square dive shape) and a maximum depth of approximately 1232 m (range: 1216–1247 m). After an IDDI of 88 min, the tagged whale made another, but shorter, benthic deep dive. The whale's location at the time of the longest dive was estimated to be at the north side of the Iceland–Faroe Ridge, at 64.1734° N 9.0855° W.

The two deepest dives in the data set started on 30 June 2016, at 15:21 UTC and 18:34 UTC, and both had the same maximum depth of approximately 2288 m (range: 2256–2319 m) (Figure 4b). The tagged whale (ID 161588, also a female/juvenile) made these dives in 3–4 km deep waters while diving downslope to the east of Jan Mayen Island, at 70.9433° N 6.6149° W and 70.9776° N 6.4692° W. The durations of the dives were 78 min and 73 min. An interval of 115 min separated the dives, but this cannot be considered an IDDI because part of the dive data was missing.

## 4 Discussion

This study described the extraordinary dive behavior of northern bottlenose whales measured using long‐duration (up to 42 days) deployments of satellite tags. We spatially limited the analyzed data to the Nordic Seas, an important area for the species, with the aim to restrict the behavioral context in which the dives were conducted. Northern bottlenose whales have been reported to migrate south to warmer waters where their dive behavior shifted to very shallow dives (Lefort et al. 2025). Overall, the patterns in diving observed here were largely consistent across individuals, with the tagged whales engaging in dives across a wide range of dive depths and dive durations. The distribution of dives was less bimodal compared to other beaked whales including the goose‐beaked whale ( *Ziphius cavirostris* ; e.g., Barlow et al. 2020; Shearer et al. 2019). The tagged northern bottlenose whales regularly dove to depths greater than 500–1000 m to presumably forage and spent very little time at the surface for gas exchange.

Individual variation in diving was also present, but limited information on the individuals excluded firm conclusions about, for example, effects of body size or age‐sex class. Northern bottlenose whales are sexually dimorphic, but the only confirmed mature male was tagged with a tag model without the extended depth range, capable of recording dive depths up to 1800–1900 m. Twenty‐one of its 149 bathypelagic dives were measured to 1848 m (the maximum depth reading for this specific tag ID) so its dive statistics in Table 2 could have been underestimated. Therefore, more prominent differences in dive behavior between the mature male and the other individuals cannot be excluded. Yet, this mature male had the highest average maximum dive depth of all the whales.

Dives as deep as 2288 m and as long as 1 h 38 min were recorded—both new records for northern bottlenose whales within the presumed natural, undisturbed context of this data set. The northern bottlenose whale appears to be among the deepest diving mammals in the world alongside the sperm whale (2000 m; Watkins et al. 1993), southern elephant seal (2388 m; Costa et al. 2010), and goose‐beaked whale (2992 m, but recorded in an area with regular sonar activity; Schorr et al. 2014). Auster and Watling (2009) interpreted seafloor gage marks around canyons and seamounts (deepest record at 2765 m) in the Northwest Atlantic to beaked whales handling or capturing prey, and similar gouges were reported for depths of up to 4258 m in the Northeast Pacific (Marsh et al. 2018). We considered 5% of the observed deep dives to be benthic (*n* = 190 dives), which we judge to be a minimum estimate due to the uncertainties associated with bathymetry and whale location. Indicative of these uncertainties is the substantial proportion of benthic dives that were deeper than the bathymetric depths that our analysis had associated with these dives (Figure S4). The maximum depths of the other, nonbenthic deep dives were also correlated with bathymetric depth, affirming the importance of the deepest parts of the ocean to this large deep‐sea predator. Such associations with the seafloor have been reported for goose‐beaked whales in the Bahamas (Joyce et al. 2017). While outside of the scope of this study, state‐space models such as hidden Markov models fitted to concurrent movement and dive observations can improve location and habitat estimates for diving animals in regions with steep bathymetric gradients (Hewitt et al. 2023).

Meso‐/bathypelagic dives accounted for 52% of the dives analyzed, and these deep dives (*n* = 3902) were performed at a rate of 1.33 per hour. Hooker and Baird (1999) reported lower values for two northern bottlenose whales tagged in the Gully, Canada, which performed 41% deep dives at a mean rate of 0.75 per hour (*n* = 57 dives). Goose‐beaked whales (*n* = 9 individuals) off Cape Hatteras, SC, USA, performed deep dives at an even lower reported mean rate of 0.43 per hour (*n* = 1408 dives) but these dives were much longer and deeper on average (Shearer et al. 2019). Differences in deep dive parameters between studies should be interpreted with care, especially in cases with methodological differences and when dive types may not serve distinct functions. Here, the depth threshold between shallow and deep dives was 200 m, but 800 m has been used for goose‐beaked whales (Baird et al. 2006, 2008; Shearer et al. 2019).

Dive type classification is a useful and widely used technique for categorizing time‐depth profiles to investigate behavioral plasticity (e.g., Shearer et al. 2022). The dives undertaken by beaked whales typically cluster into two or three distinct dive types, including those of Baird's ( *Berardius bairdii* ; Minamikawa et al. 2007), goose‐ (Schorr et al. 2014; Cioffi et al. 2021) and Blainville's beaked whale (Arranz et al. 2011). Similarly, three clusters were used to categorize the dives of northern bottlenose whales off Jan Mayen into types based on short‐term, high‐resolution DTAG data, although the clustering was not very strong (Siegal 2020; Siegal et al. 2022). Siegal (2020) named these three dive types, which are roughly comparable to those used here, *short/shallow*, *mid‐depth*, and *long/deep*. Two clusters better separated the dives of northern bottlenose whales in the Gully (Hooker and Baird 1999; Siegal 2020). In this study, we used an informed categorization based on biologically meaningful dive depth categories instead of an unsupervised approach such as k‐means clustering, and the degree of clustering on the two dimensions of dive depth and duration was relatively weak (Figure 3). Several tag records included series of consecutive deep dives that were almost exclusively mesopelagic (e.g., Figure S8). Conceivably, these could represent a different type of foraging dive than single deep dives separated by longer IDDIs. An analysis that considers temporal correlation might be a more powerful approach to differentiate between these two foraging periods.

Long and deep dives of beaked whales are usually foraging dives, whereas short and shallow dives likely serve various functions including socializing, foraging, traveling, and respiration (Hooker et al. 2009; Siegal et al. 2022; Tyack et al. 2006). Interpretations of (baseline) dive records should also always consider the possibility that a small fraction of dives, presumably the more extreme ones, in fact may entail behavioral responses such as conspicuously long and deep dives to a natural or human stressor. Silent dives, that is, those without production of echolocation clicks or other sounds, may serve to abate predation risk (real or perceived) induced by actual predator presence or human activities like naval sonar (Aguilar de Soto et al. 2020; Miller et al. 2022). For example, the correlations between the top 5% of dive durations and their preceding or following IDDI durations may have been affected by such underlying motivations of deep diving behavior.

The main prey of northern bottlenose whales in the Nordic Seas is the abundant Boreoatlantic armhook squid *Gonatus fabricii*, which descends to deeper depths with each ontogenetic stage (Benjaminsen and Christensen 1979; Bjørke 2001; Golikov et al. 2018). Epi‐ to bathypelagic sizes of *Gonatus* spp. (Arkhipkin and Bjørke 1999) have been found in the stomach contents of individuals that stranded in the Northeast Atlantic (Fernández et al. 2014; Santos et al. 2001; Marine and Freshwater Research Institute, unpublished data). Most deep dives recorded here were presumably foraging dives. We can only speculate on the function of the epipelagic dives, but we acknowledge that they likely also included some foraging dives. Buzz sounds, indicating foraging attempts, by northern bottlenose whales have been previously documented within DTAG data throughout the water column but mainly at depths below 100–300 m (Figure 4 in Miller et al. 2015; Haas et al. 2025). Given their demonstrated capacity for conducting long and deep dives (Hooker et al. 2009; Joyce et al. 2017; Quick et al. 2020; Tyack et al. 2006), beaked whale foraging depths might be motivated by prey availability and type on a dive‐by‐dive basis (Arranz et al. 2011). Hence, northern bottlenose whales, like sperm whales (Watwood et al. 2006), could be exploiting different depths depending on the abundance of their squid prey. Also, being a larger and gregarious species of beaked whale, they may have a more relaxed acoustic crypsis (Madsen et al. 2014), so there could be a lower predation risk to producing echolocation at relatively shallower depths in this species.

Our analysis was only conducted on the lower‐resolution dive summary data because the sample size of the higher‐resolution depth data was considered too small to provide robust results; the cumulative duration of the higher‐resolution data was only 12.5 days while that of the lower‐resolution data was 132.5 days. In addition, visual inspection of the dive profiles (Figures S5–S16) suggested a high degree of concurrence between the two types of dive data. Future use of such higher‐resolution data from satellite tags, or the use of custom onboard processing of, for example, accelerometer data to detect foraging events (Siegal 2020), is likely to increase our ability to differentiate functionally different dives in northern bottlenose whales. Considering only the vertical component of movement may also limit the interpretation of the data as well as statistical power to detect complex patterns. Combining vertical and horizontal movement, as well as controlling for individual, social, and environmental factors, in future analyses could offer new information on dive functions, behavior plasticity, and habitat use in this species.

This study advanced our knowledge of the extreme dive behavior of northern bottlenose whales in the Northeast Atlantic and highlights the importance of their foraging habitat around Jan Mayen, the Faroe Islands, and Iceland. Human offshore activities in the Nordic Seas, such as heavy shipping and deep‐sea mining, are projected to increase (Gilbert 2024; PAME 2024; Thompson et al. 2023), with potential consequences for the predators that forage in the deepest depths of our oceans. The limited knowledge of deep‐diving beaked whales and their prey makes assessing the potential effects of threats such as noise pollution, fisheries interactions, and climate change on them difficult, let alone thinking about preventive conservation measures (Feyrer, Stanistreet, and Moors‐Murphy 2024; Hooker et al. 2019).

## Author Contributions

**Barbara K. Neubarth:** conceptualization (equal), formal analysis (equal), visualization (lead), writing – original draft (lead), writing – review and editing (equal). **Patrick J. O. Miller:** data curation (supporting), funding acquisition (equal), project administration (supporting), writing – review and editing (supporting). **Rune Roland:** data curation (equal), funding acquisition (supporting), writing – review and editing (supporting). **Lars Kleivane:** data curation (supporting), writing – review and editing (supporting). **Paul J. Wensveen:** conceptualization (equal), data curation (equal), formal analysis (equal), funding acquisition (equal), project administration (lead), supervision (lead), writing – review and editing (equal).

## Conflicts of Interest

The authors declare no conflicts of interest.

## Supporting information


**Data S1:** ece371862‐sup‐0001‐Supinfo.pdf.

## Data Availability

All relevant data and code are within the paper and its electronic Supporting Information, or are available from GitHub at https://github.com/PaulWens/bottlenose‐whale‐diving.
