# Supplementary material for: Long‐Term Depth Records of Satellite‐Tagged Northern Bottlenose Whales Reveal Extraordinary Dive Capabilities
Source: Ecol Evol. 2025 Jul 30;15(8):e71862. doi: 10.1002/ece3.71862 (PMC12310271; doi:10.1002/ece3.71862)
Supplement: Supplementary file 1 — Data S1: ece371862‐sup‐0001‐Supinfo.pdf. [file ECE3-15-e71862-s001.pdf]

Supplementary materials

Table S1. Overview of the models and settings of the depth-transmitting satellite tags used in this study. Note that the tags defined a dive in the Behavior Log using a threshold of 10 or 20 m; however, dives to less than 40 m were added to the surface periods in post-processing, for consistency with previous work (Hooker and Baird, 1999; Siegal, 2020). The deployments are listed in chronological order.

Tag ID / Argos PTT	Year	Tag model	Argos repetition interval	Re-transmit over period	Behavior Log settings				Time Series settings	
					Sampling interval	Surface	Min depth	Min duration	Sampling interval	Duty cycle
134664	2014	SPLASH10-292B	44 s	2 days	10 s	Wet/dry	20 m	1 min	2.5 min	5 days off, 1 day on
134666	2014	SPLASH10-292B	46 s	3 days	10 s	Wet/dry	20 m	1 min	2.5 min	5 days off, 1 day on
134663	2014	SPLASH10-292A	45 s	3 days	10 s	Wet/dry	10 m	1 min	2.5 min	5 days off, 1 day on
134670	2015	SPLASH10-292B	15 s	2 days	10 s	Wet/dry	20 m	1 min	2.5 min	5 days off, 1 day on
134669	2015	SPLASH10-292B	15 s	2 days	10 s	Wet/dry	20 m	1 min	2.5 min	5 days off, 1 day on
134668	2015	SPLASH10-292A	15 s	2 days	10 s	Wet/dry	20 m	1 min	2.5 min	5 days off, 1 day on
161587	2016	SPLASH10-292B	15 s	4 days	1 s	5 m	20 m	1 min	2.5 min	6 days off, 1 day on
161588	2016	SPLASH10-292B	15 s	4 days	1 s	5 m	20 m	1 min	2.5 min	6 days off, 1 day on
161590	2016	SPLASH10-292B	15 s	4 days	1 s	Wet/dry	20 m	1 min	2.5 min	6 days off, 1 day on
161592	2016	SPLASH10-292B	15 s	4 days	1 s	Wet/dry	20 m	1 min	2.5 min	6 days off, 1 day on
161593	2016	SPLASH10-292B	15 s	4 days	1 s	5 m	20 m	1 min	2.5 min	6 days off, 1 day on
161591	2016	SPLASH10-292B	15 s	4 days	1 s	5 m	20 m	1 min	2.5 min	6 days off, 1 day on
134667	2016	SPLASH10-292A	15 s	4 days	1 s	Wet/dry	10 m	1 min	2.5 min	6 days off, 1 day on

Figure S1. Duration of the top 5% of deep dives in 2016 (longer than 58.7 min) as function of the inter-deep dive interval (left) before and (right) after the dive. Note that IDDI is plotted on a log-scale.

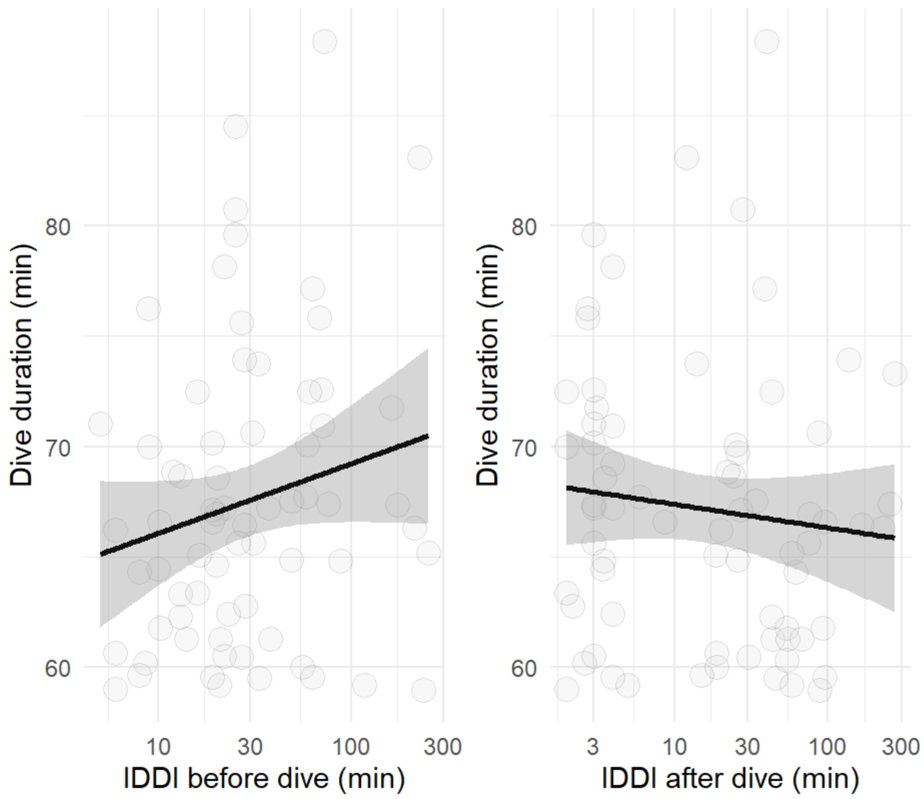

Figure S2. Maximum depth and duration of dives recorded in 2016, colored by tag ID.

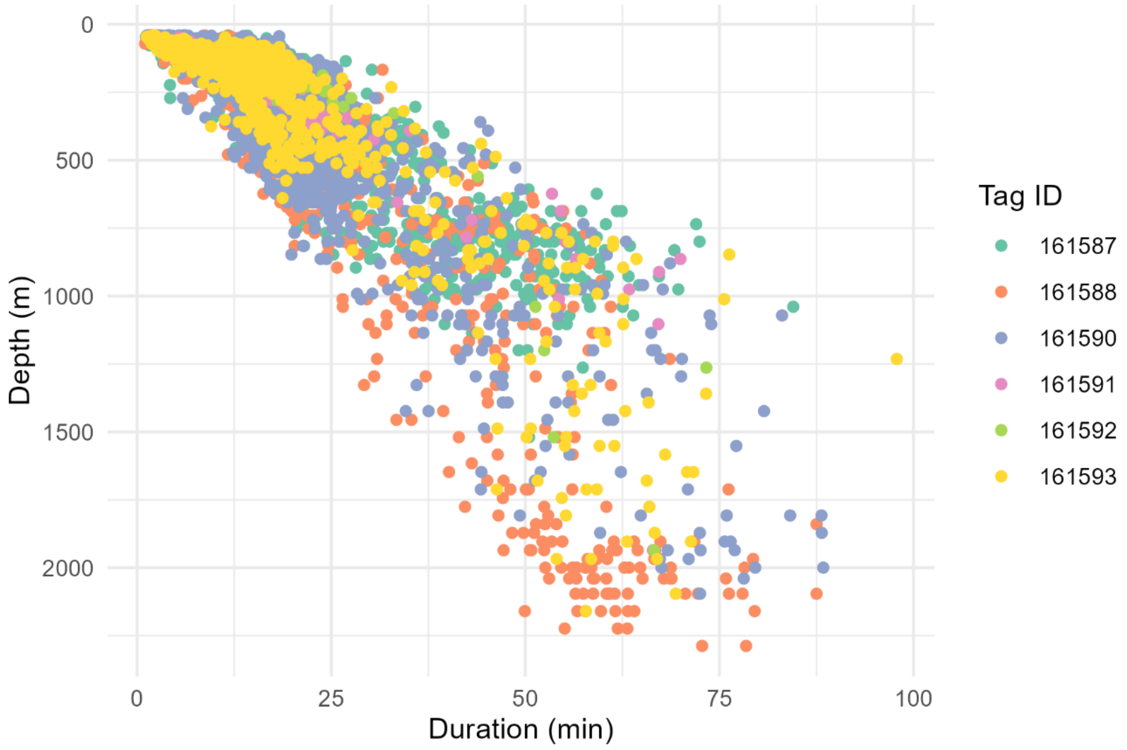

Figure S3. Scatterplot of the geographical coordinates of the recorded dives (grey circles) and their marginal distribution plots. The coordinates of the island Jan Mayen (green triangle), a line along the middle of the Iceland-Faroes Ridge (dashed line) and geographical contours are indicated as reference. Dives that were conducted above and south of the Iceland-Faroe Ridge were not included in the analysis because the whales were in a different behavioral state.

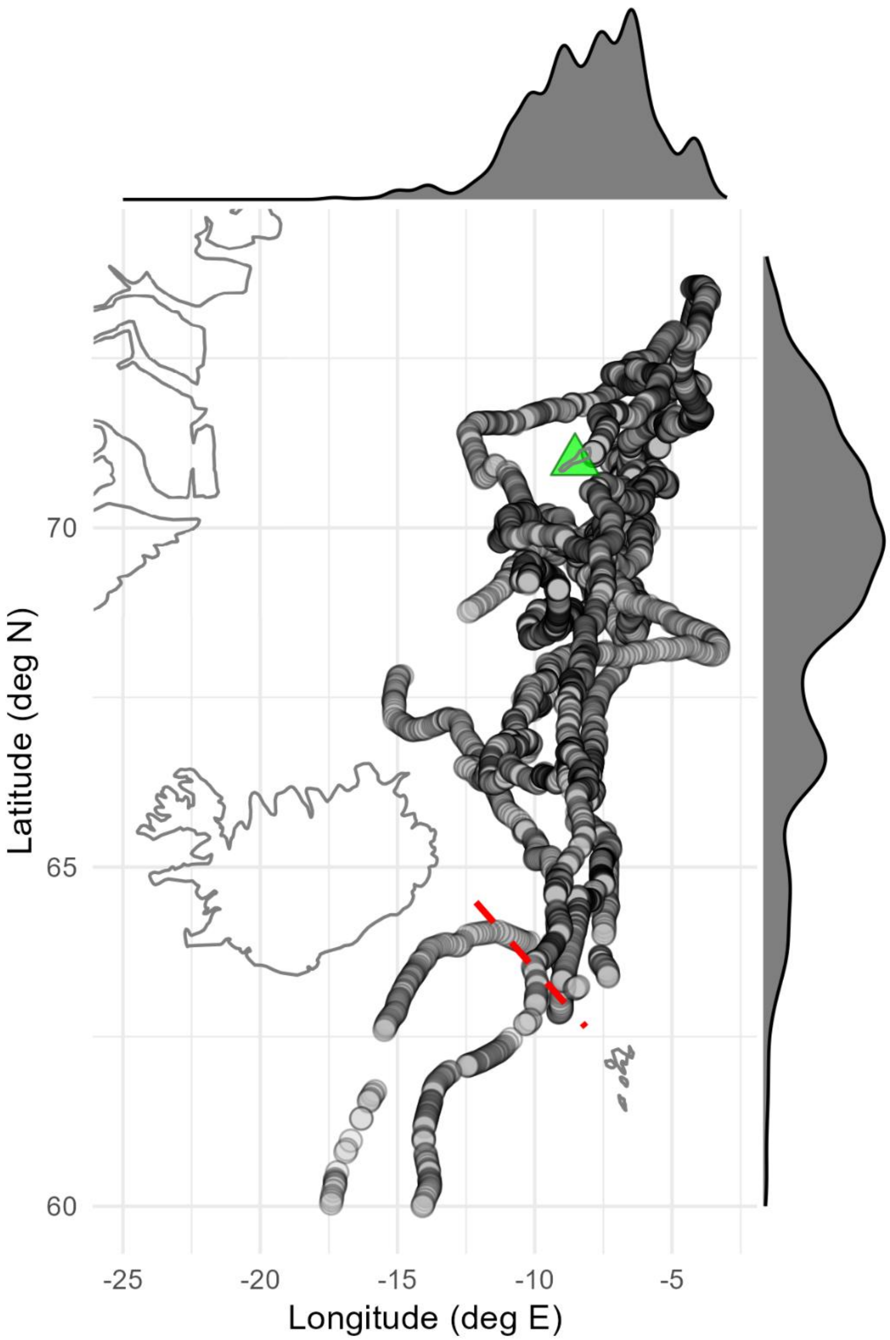

Figure S4. Depth distribution of benthic dives (defined as dives with a maximum depth deeper than 100 m shallower than the charted seafloor depth for that location) relative to the charted depth of the seafloor. Positive values represent dive depths that were greater than the charted seafloor depth at the dive location.

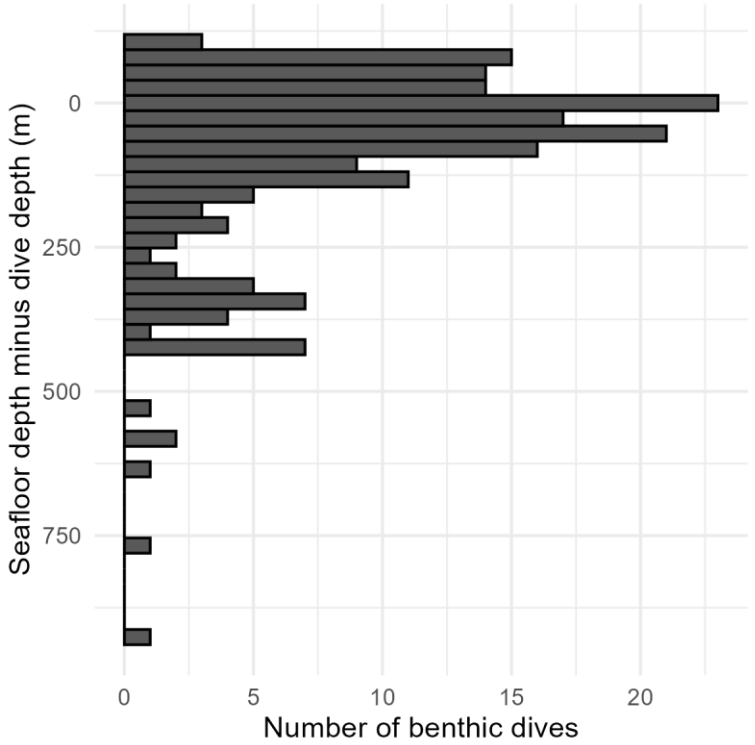

Figures S5-16. Dive profiles per tag reconstructed from dive summary (black lines) and time series (orange lines) information. Turquoise dots: seafloor depth; red asterisks: dives considered benthic (< 100 m from the seafloor or beyond charted seafloor depths). Dashed vertical lines mark time of tagging or intentional sonar exposure. Dive summaries of 2014-2015 (Figures S5-9) include a small, but unknown number of undetected surfaces and are thus, not a totally accurate representation of the whales' dive behavior.

Figure S5.

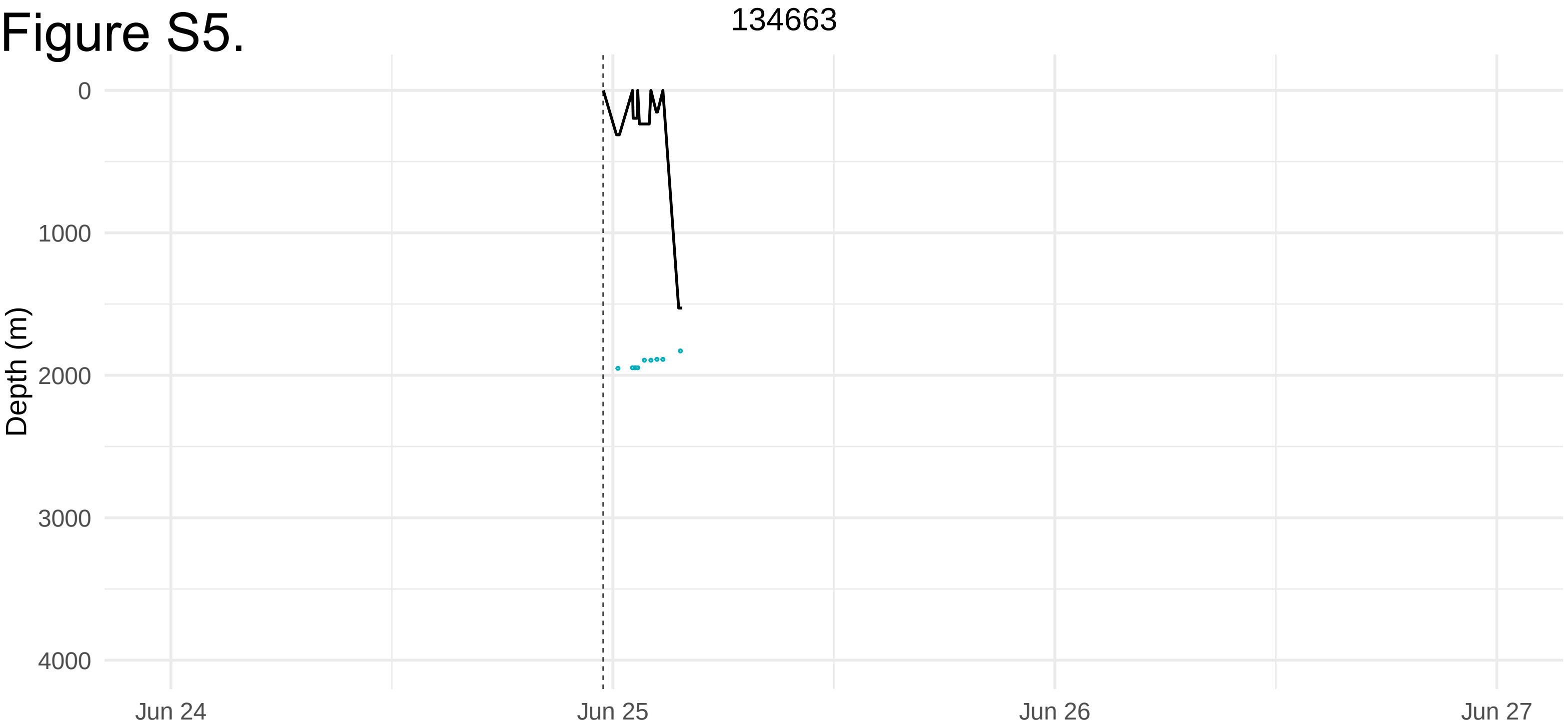

Figure S6.

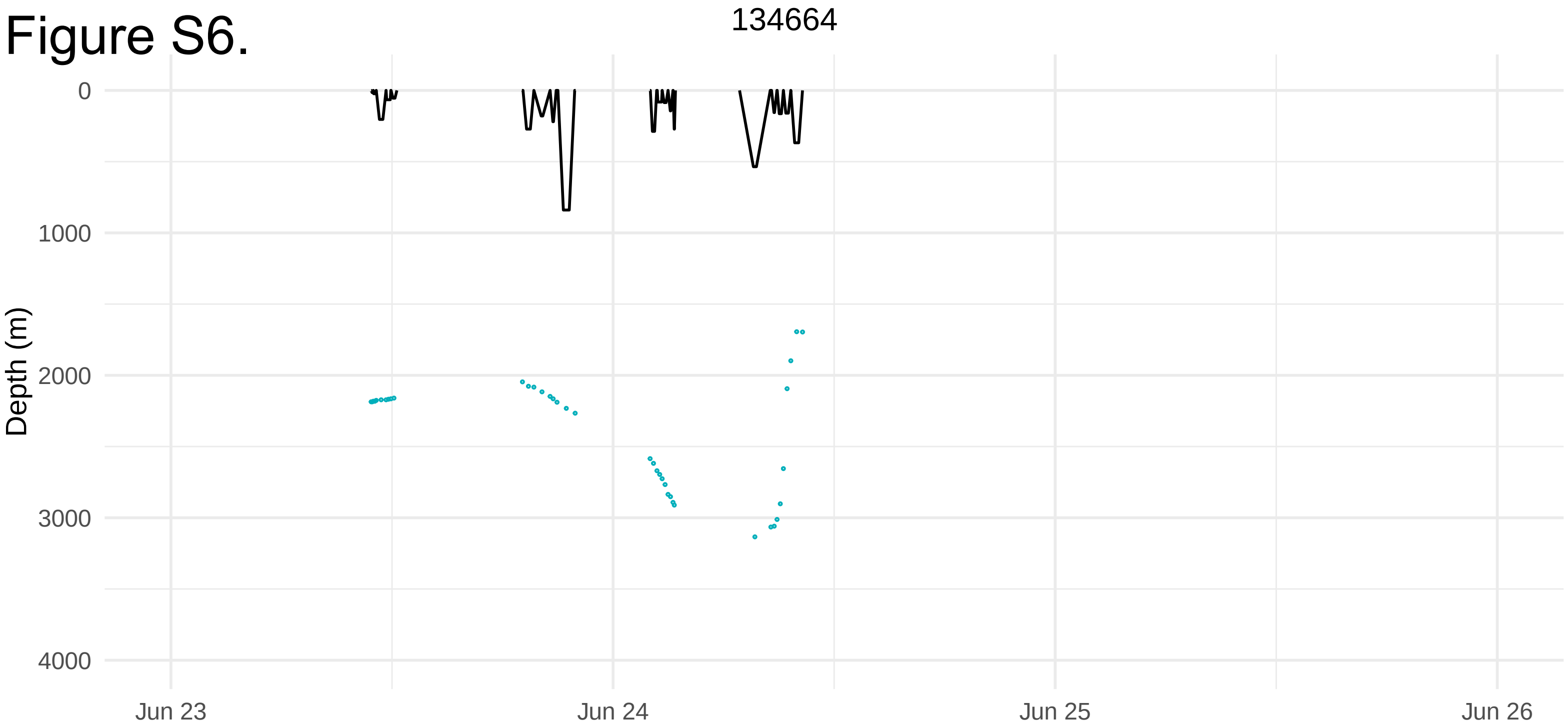

Figure S7.

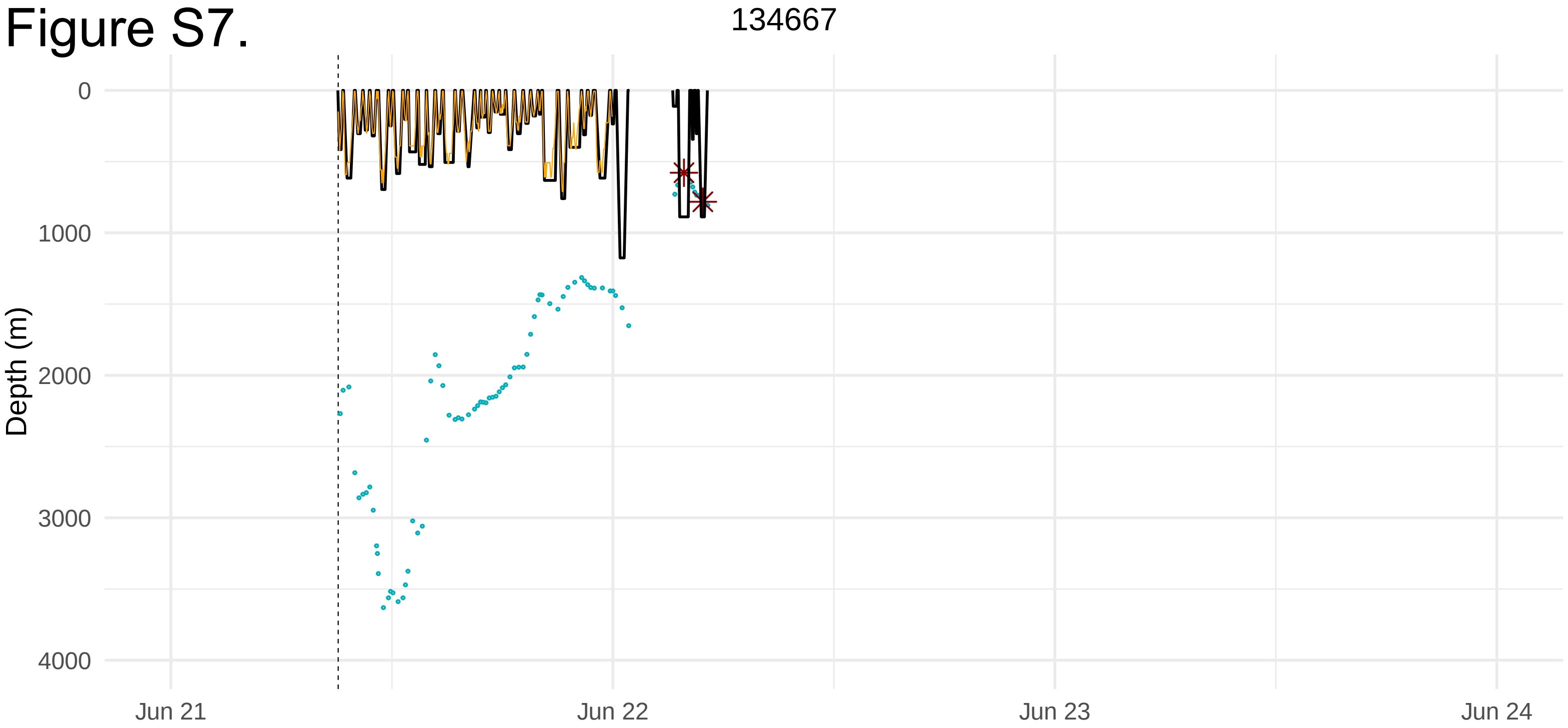

Figure S8.

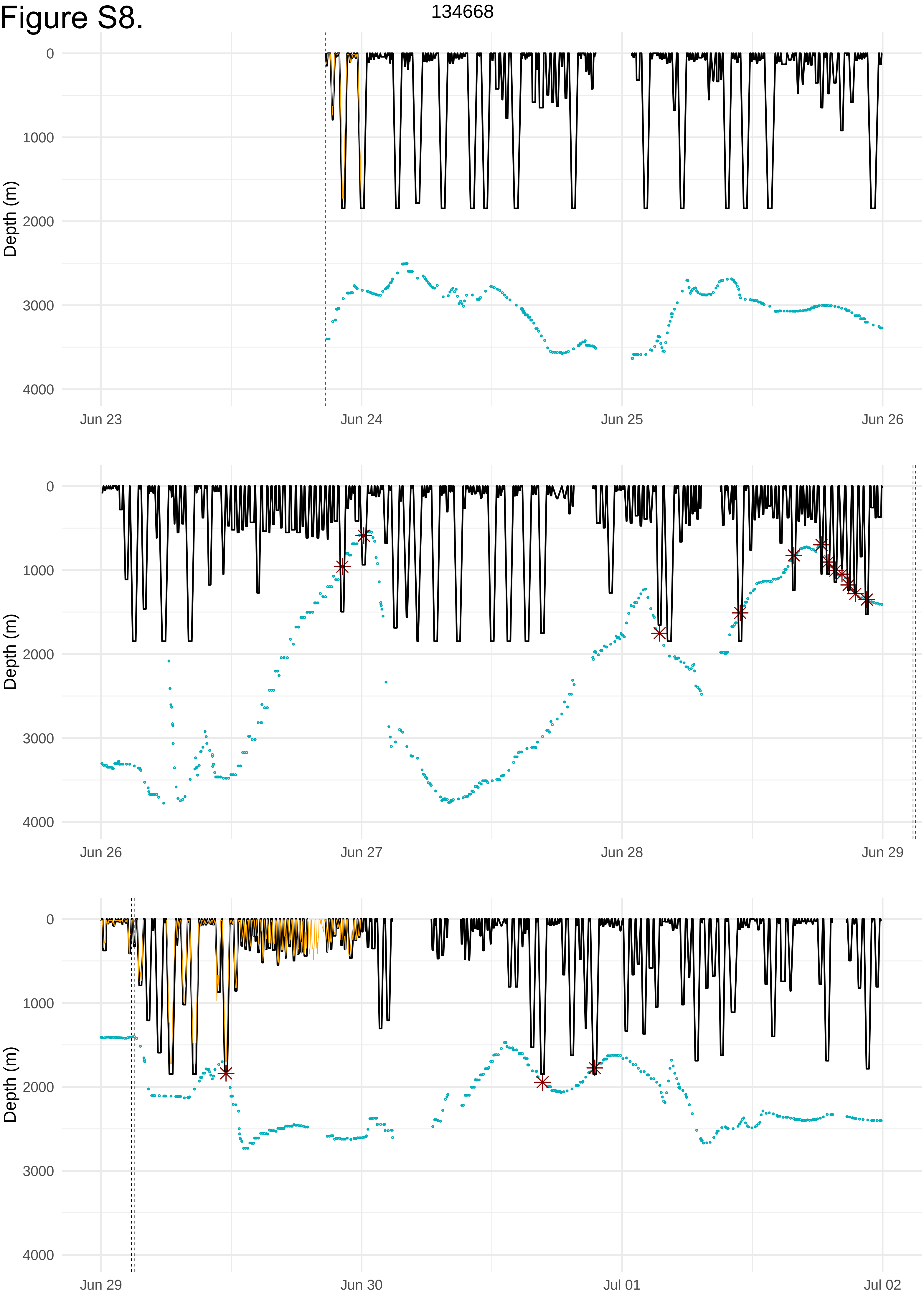

134668
Depth (m)
0
1000
2000
3000
4000
Jun 23
Jun 24
Jun 25
Jun 26
Jun 27
Jun 28
Jun 29
Jun 30
Jul 01
Jul 02

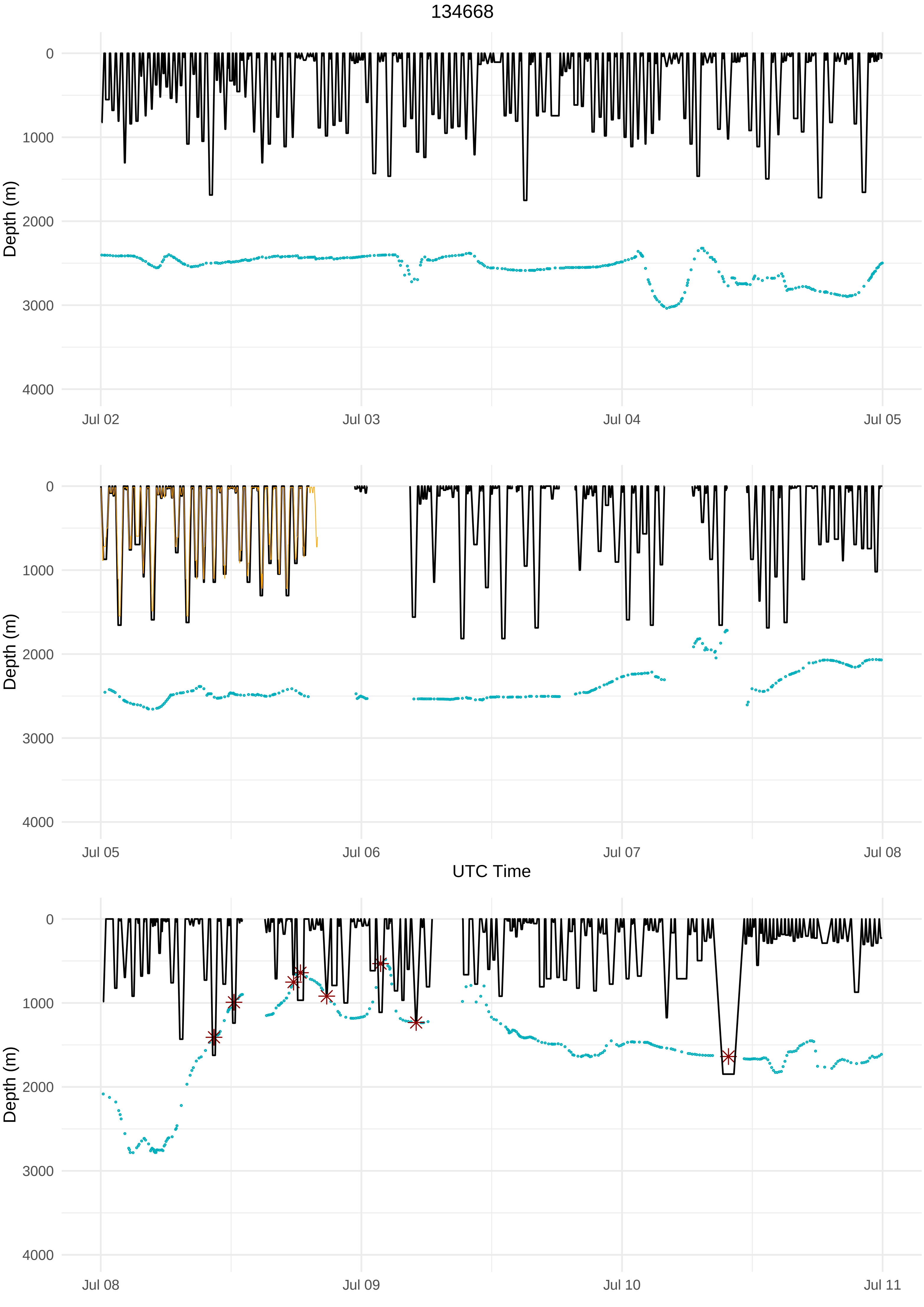
134668
Depth (m)
0
1000
2000
3000
4000
Jul 02
Jul 03
Jul 04
Jul 05
Jul 06
Jul 07
Jul 08
UTC Time
Jul 09
Jul 10
Jul 11

134668

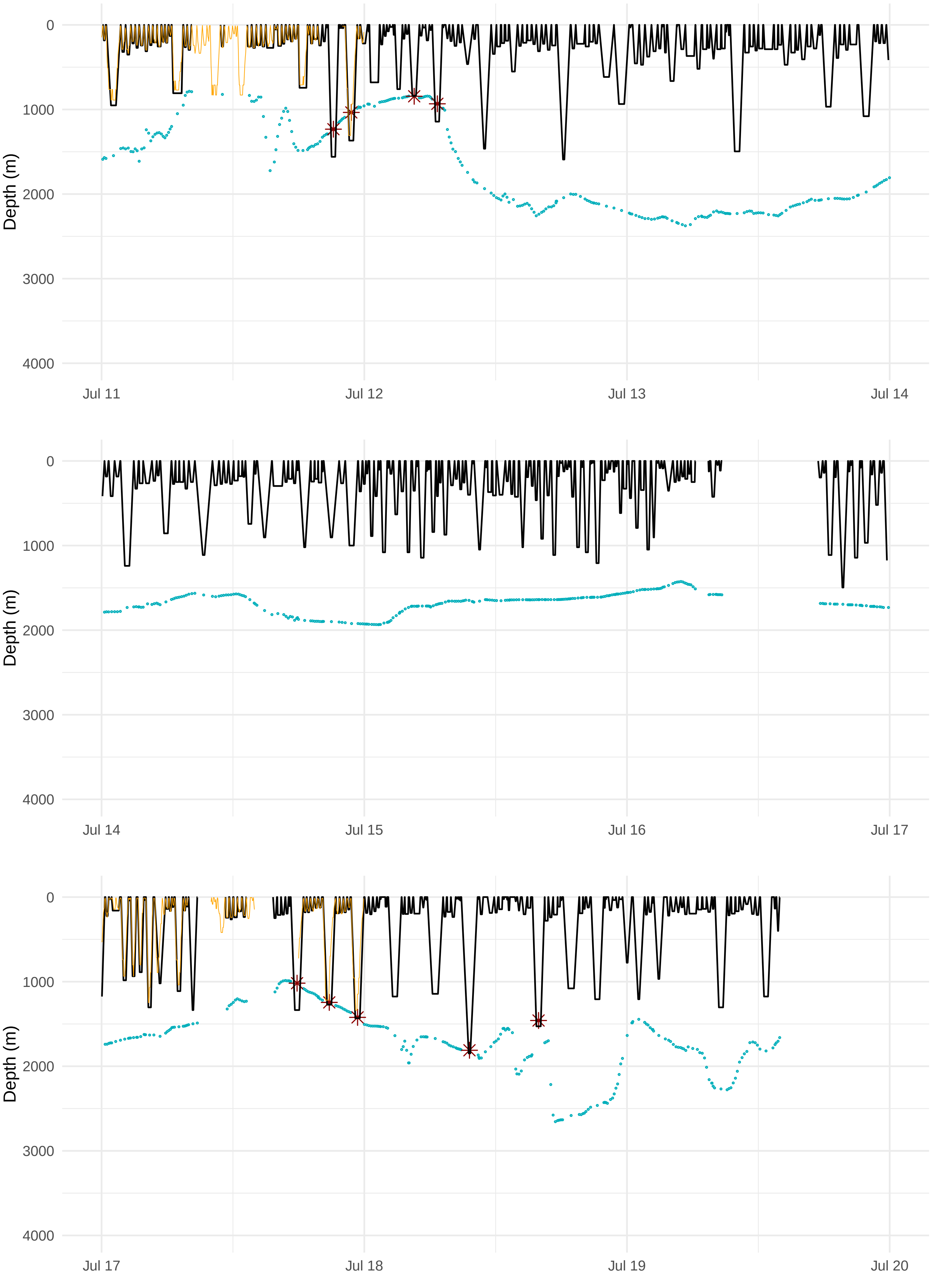

Depth (m)
0
1000
2000
3000
4000
Jul 11
Jul 12
Jul 13
Jul 14
Jul 15
Jul 16
Jul 17
Jul 18
Jul 19
Jul 20

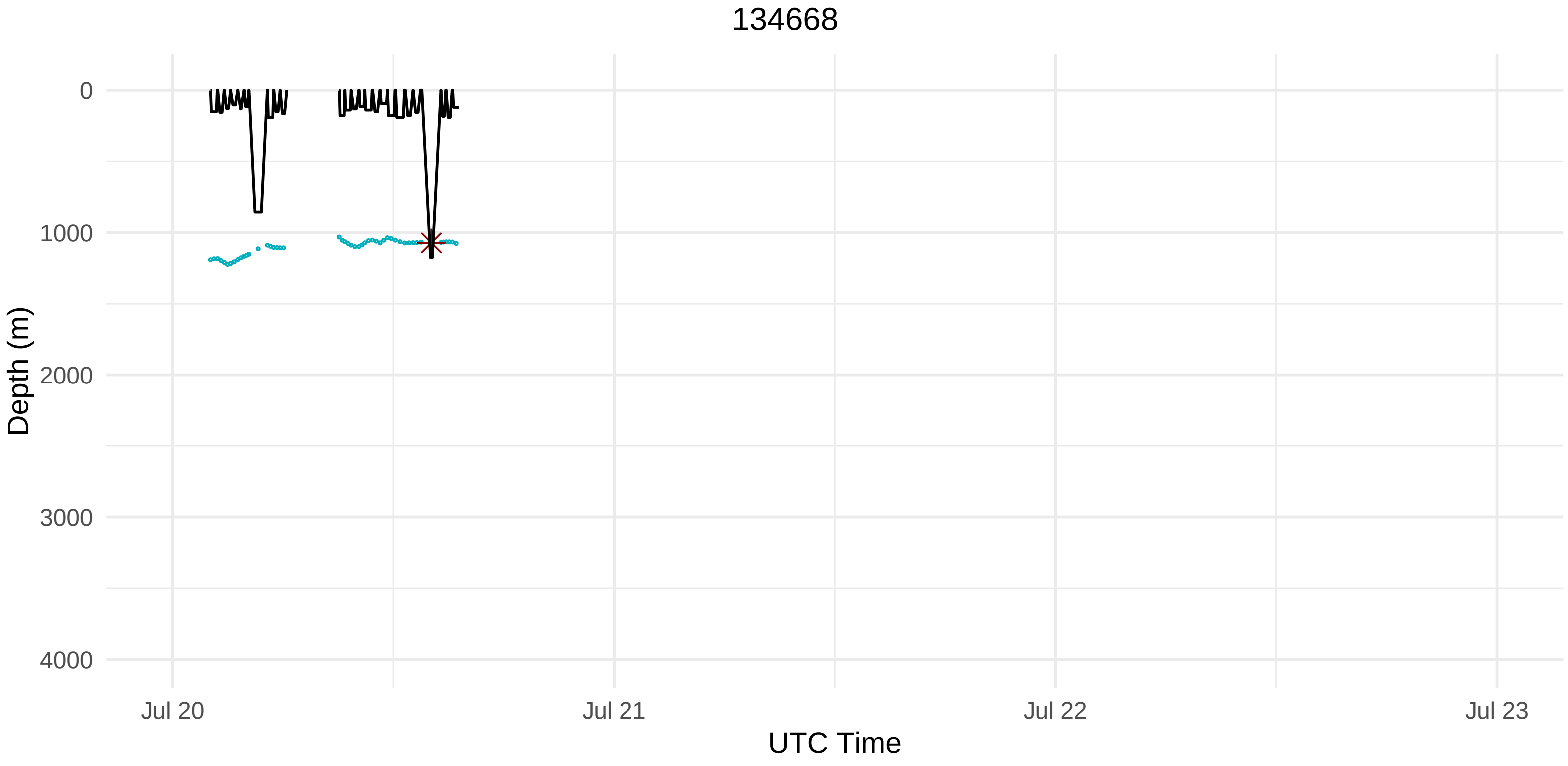

134668
0
1000
2000
3000
4000
Depth (m)
Jul 20
Jul 21
Jul 22
Jul 23
UTC Time

Figure S9.

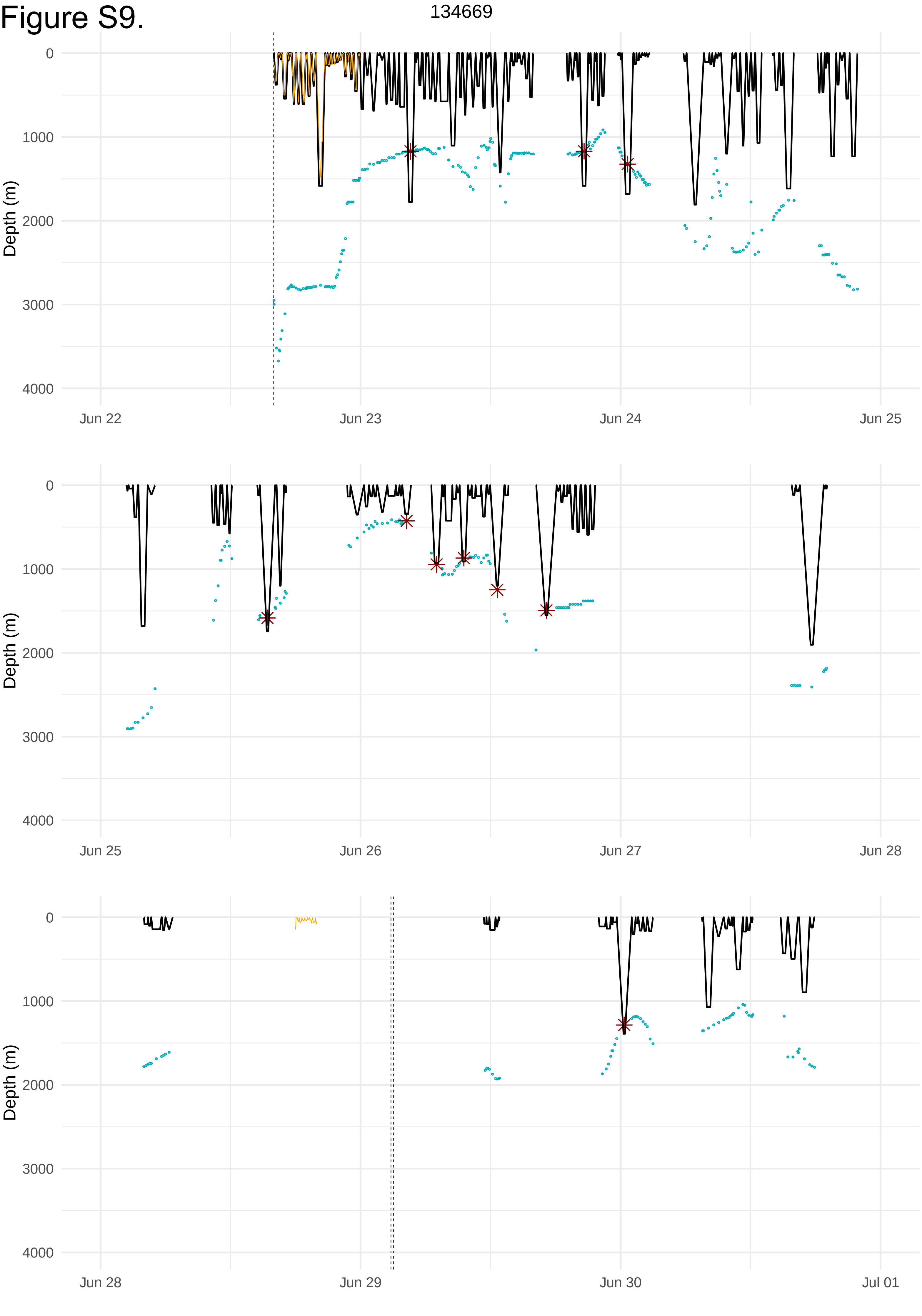
134669
Depth (m)
0
1000
2000
3000
4000
Jun 22
Jun 23
Jun 24
Jun 25
Jun 26
Jun 27
Jun 28
Jun 29
Jun 30
Jul 01

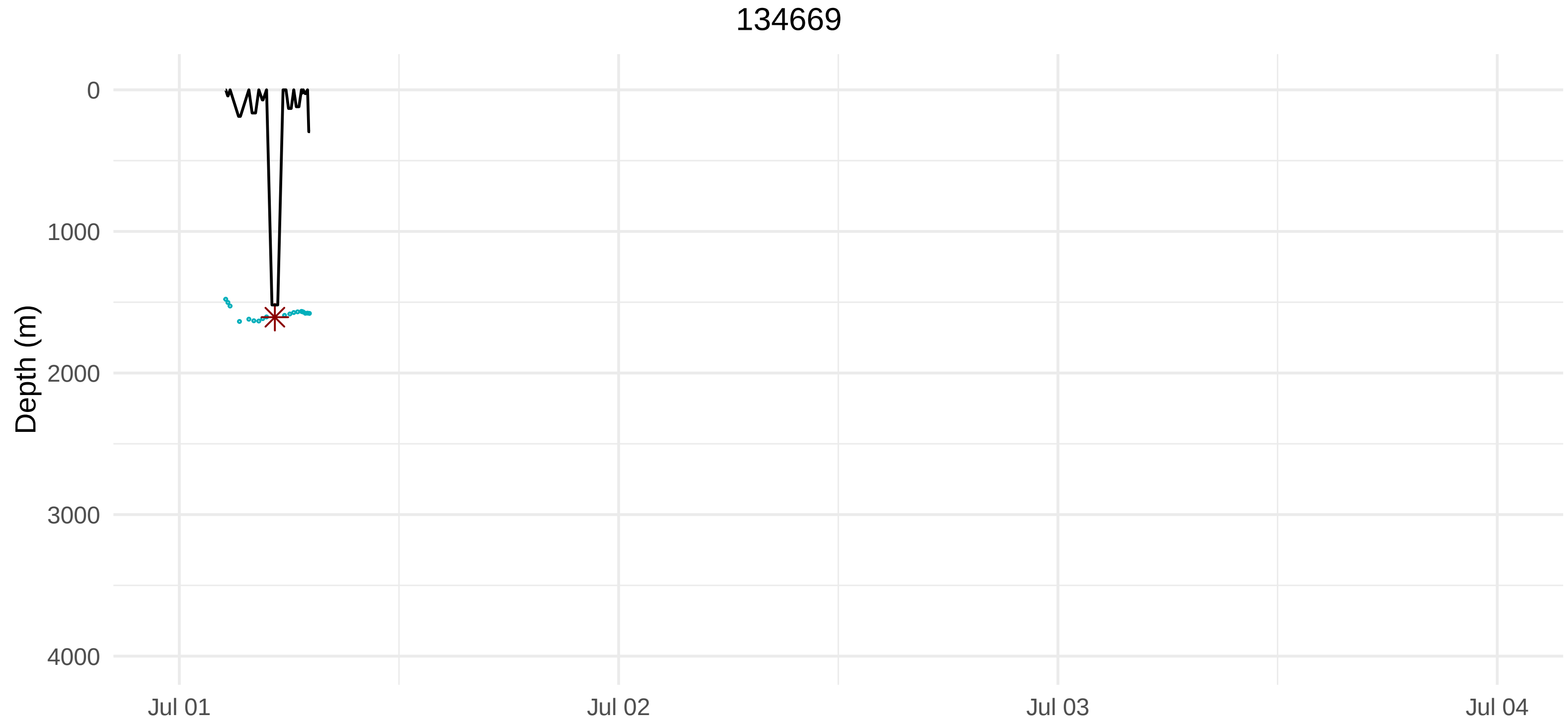

134669
0
1000
2000
3000
4000
Depth (m)
Jul 01
Jul 02
Jul 03
Jul 04

Figure S10.

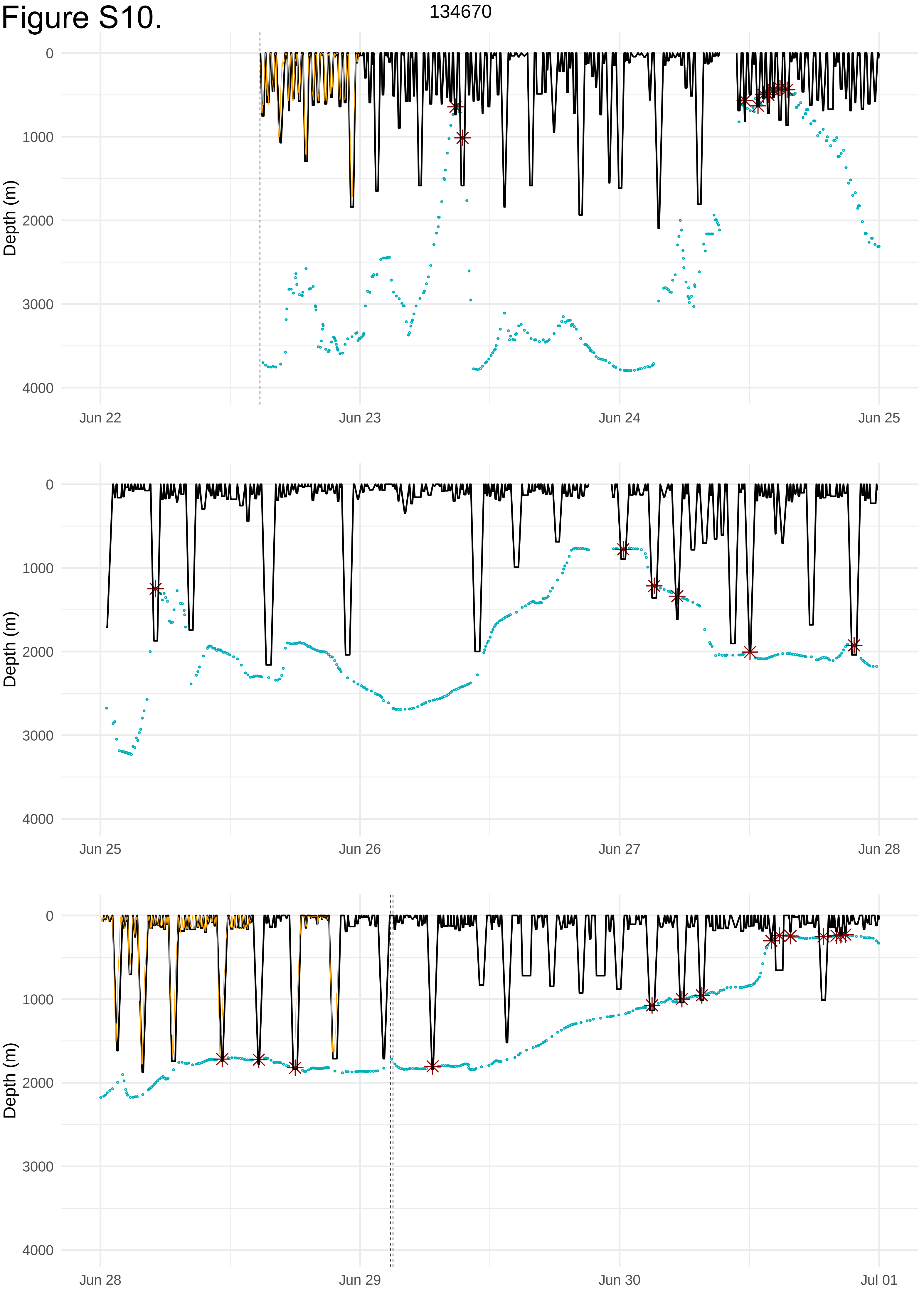

134670
Depth (m)
0
1000
2000
3000
4000
Jun 22
Jun 23
Jun 24
Jun 25
Jun 26
Jun 27
Jun 28
Jun 29
Jun 30
Jul 01

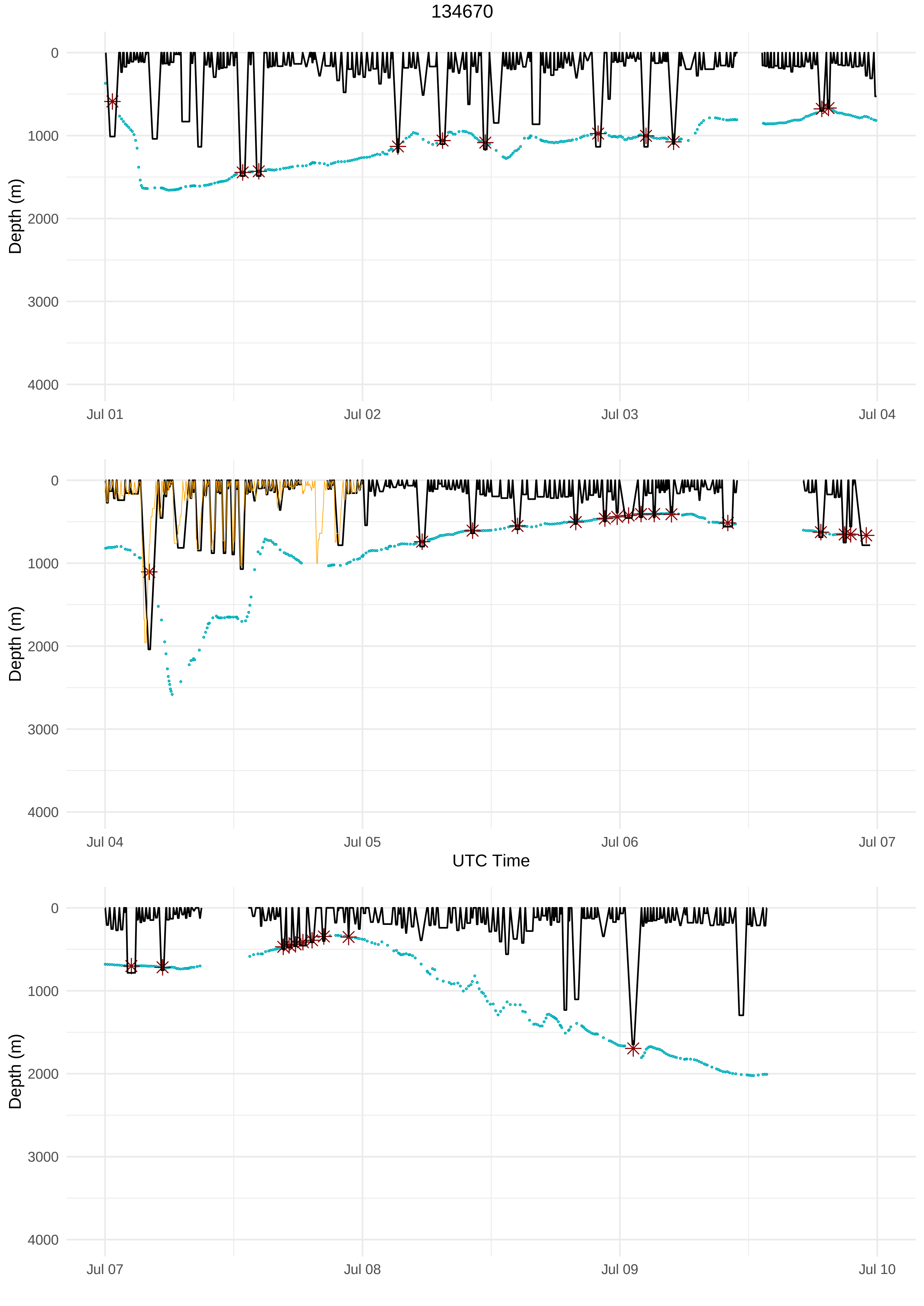

134670
Depth (m)
0
1000
2000
3000
4000
Jul 01
Jul 02
Jul 03
Jul 04
Jul 05
Jul 06
Jul 07
UTC Time
Jul 08
Jul 09
Jul 10

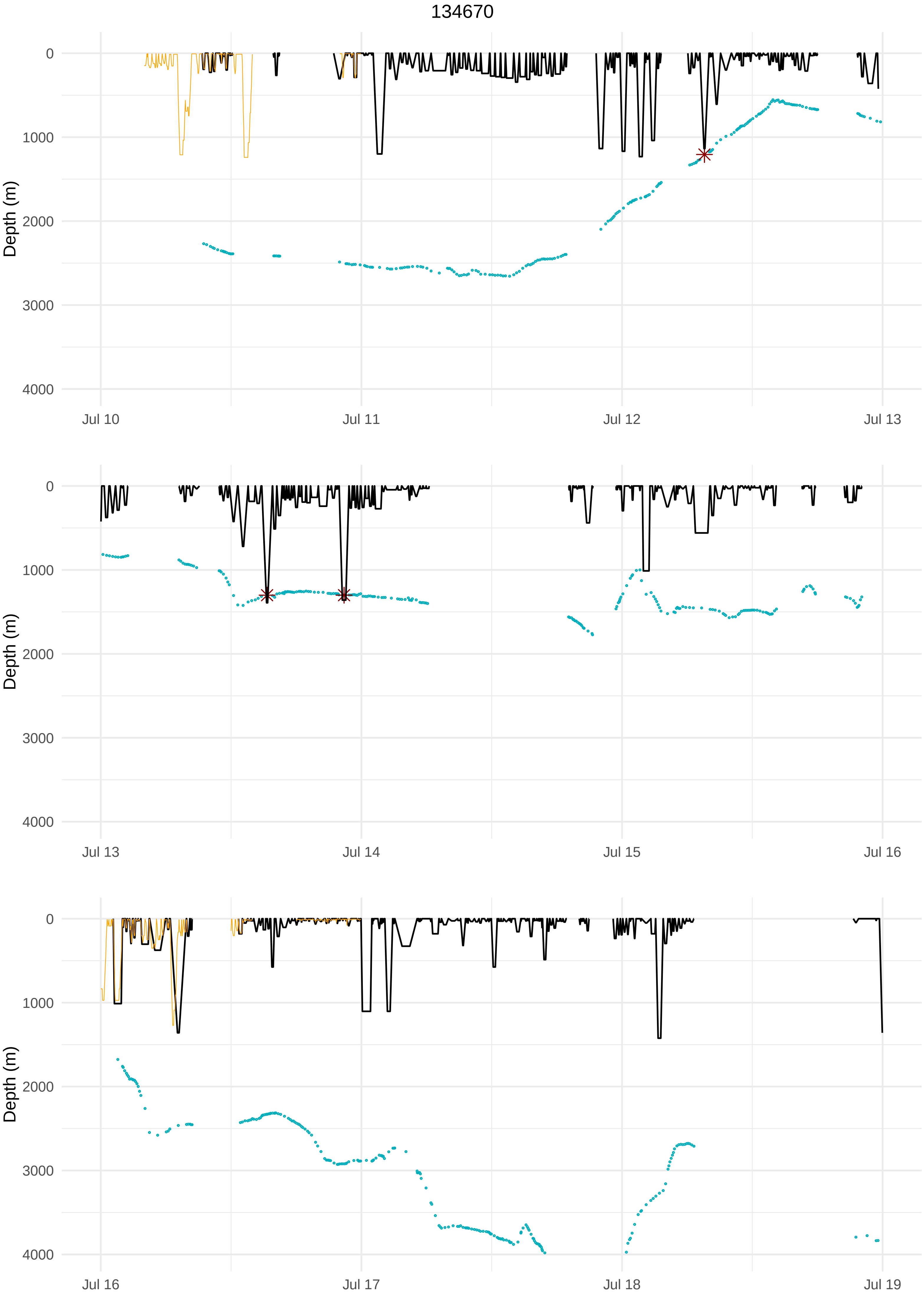

134670
Depth (m)
0
1000
2000
3000
4000
Jul 10
Jul 11
Jul 12
Jul 13
Depth (m)
0
1000
2000
3000
4000
Jul 13
Jul 14
Jul 15
Jul 16
Depth (m)
0
1000
2000
3000
4000
Jul 16
Jul 17
Jul 18
Jul 19

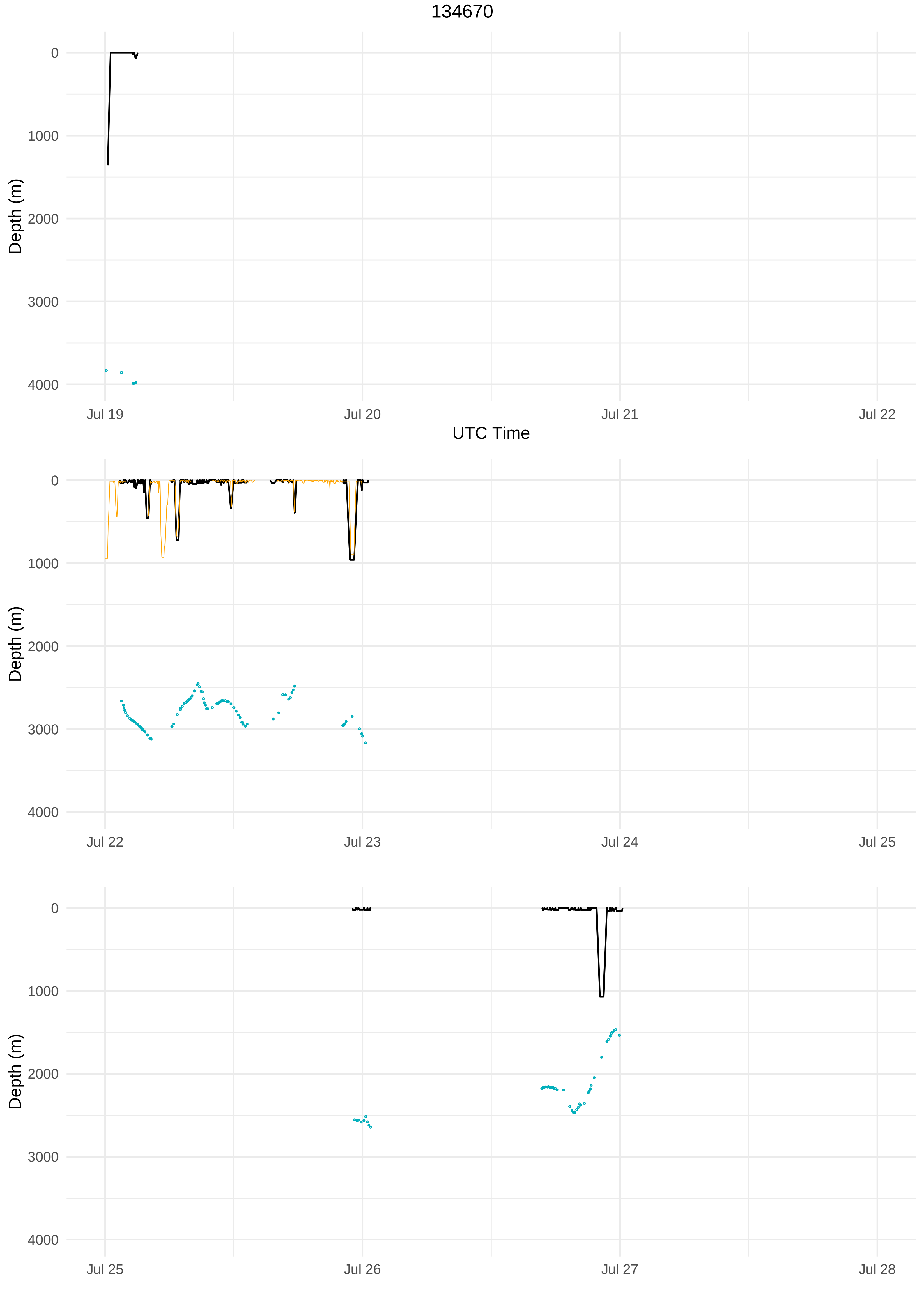
134670
0
1000
2000
3000
4000
Depth (m)
Jul 19
Jul 20
Jul 21
Jul 22
UTC Time
0
1000
2000
3000
4000
Depth (m)
Jul 22
Jul 23
Jul 24
Jul 25
0
1000
2000
3000
4000
Depth (m)
Jul 25
Jul 26
Jul 27
Jul 28

134670

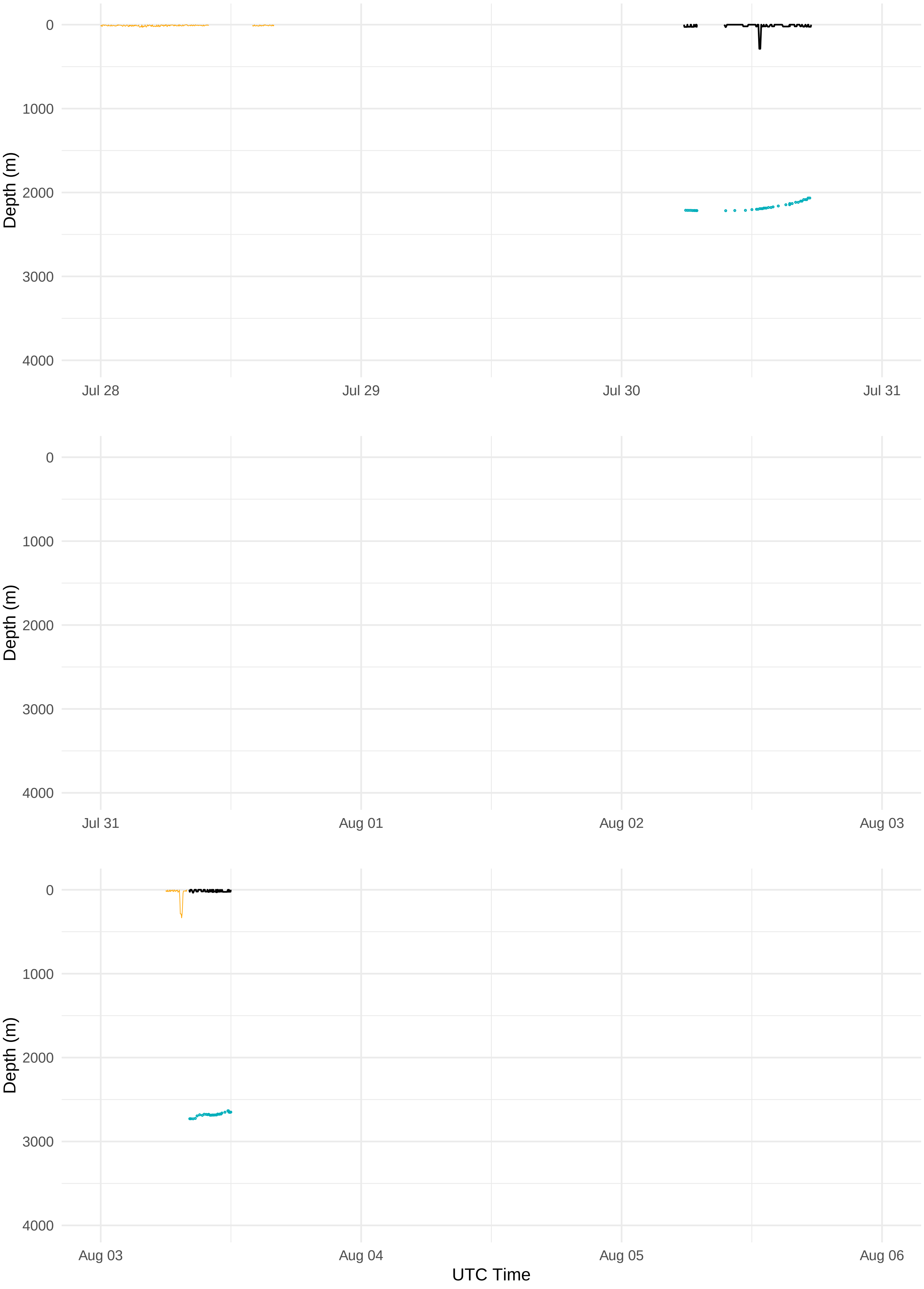

0
1000
2000
3000
4000
Depth (m)
Jul 28
Jul 29
Jul 30
Jul 31
0
1000
2000
3000
4000
Depth (m)
Jul 31
Aug 01
Aug 02
Aug 03
0
1000
2000
3000
4000
Depth (m)
Aug 03
Aug 04
Aug 05
Aug 06
UTC Time

Figure S11.

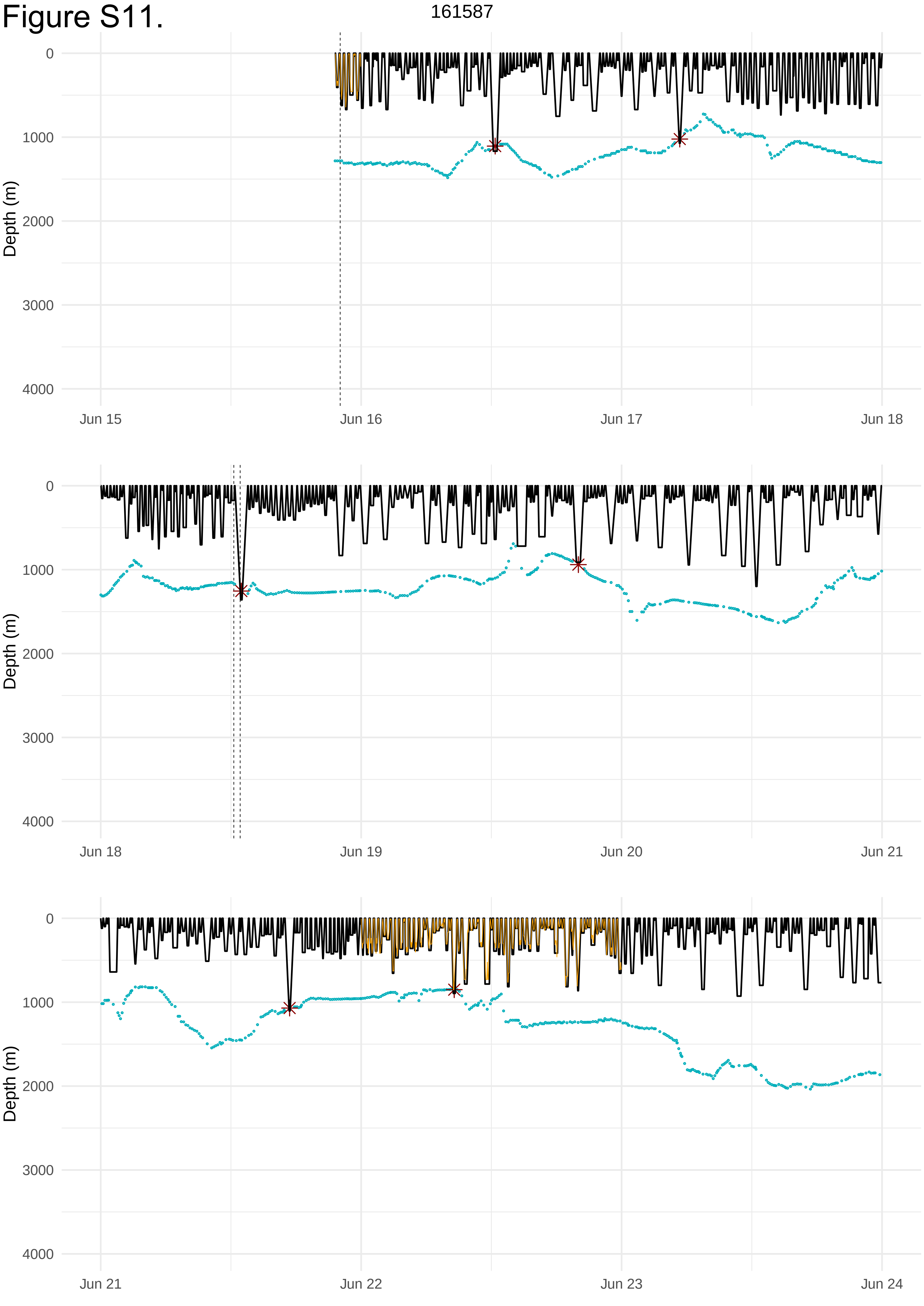
161587
Depth (m)
0
1000
2000
3000
4000
Jun 15
Jun 16
Jun 17
Jun 18
Depth (m)
0
1000
2000
3000
4000
Jun 18
Jun 19
Jun 20
Jun 21
Depth (m)
0
1000
2000
3000
4000
Jun 21
Jun 22
Jun 23
Jun 24

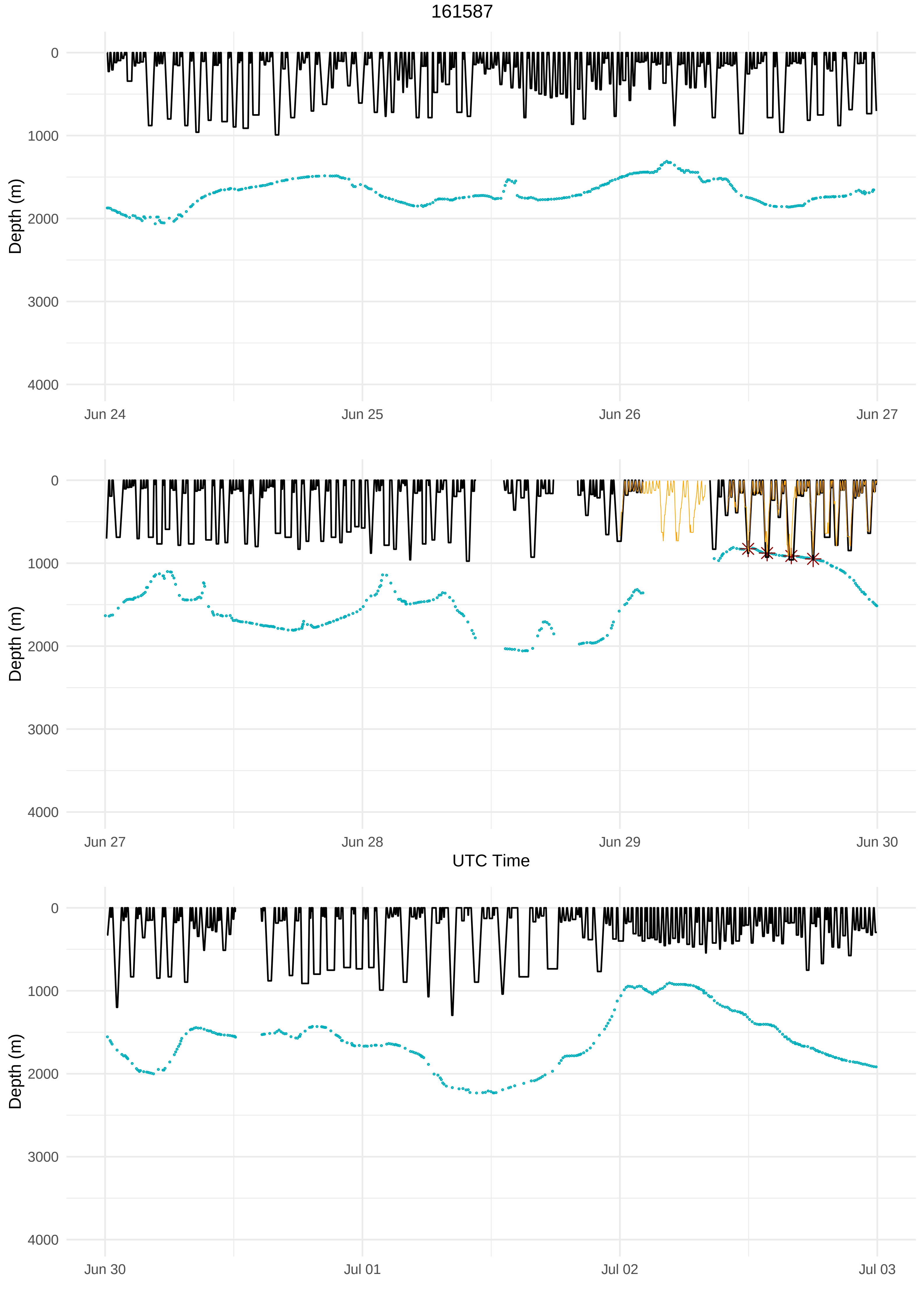

161587
Depth (m)
0
1000
2000
3000
4000
Jun 24
Jun 25
Jun 26
Jun 27
Depth (m)
0
1000
2000
3000
4000
Jun 27
Jun 28
Jun 29
Jun 30
UTC Time
Depth (m)
0
1000
2000
3000
4000
Jun 30
Jul 01
Jul 02
Jul 03

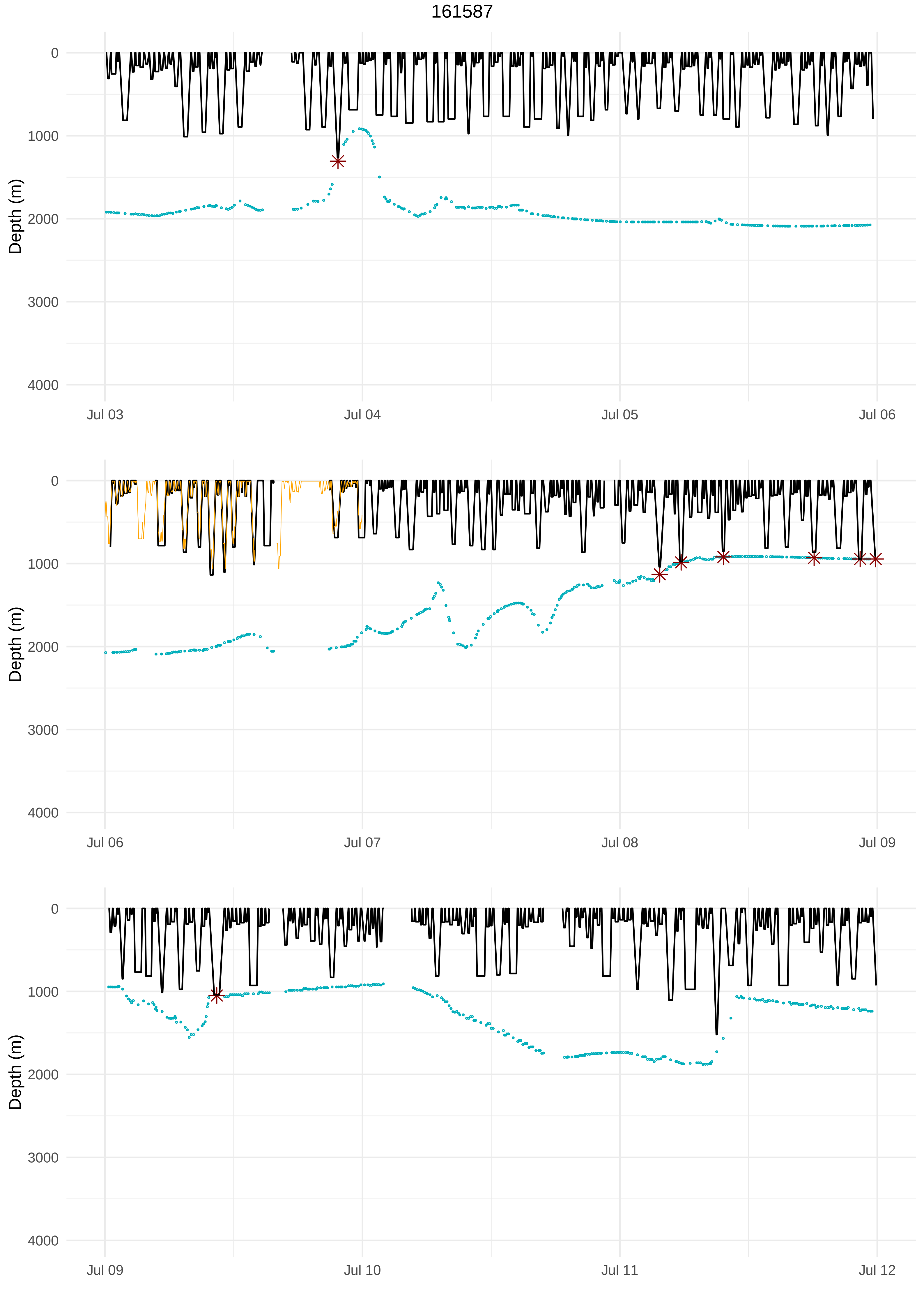
161587
Depth (m)
0
1000
2000
3000
4000
Jul 03
Jul 04
Jul 05
Jul 06
Jul 07
Jul 08
Jul 09
Jul 10
Jul 11
Jul 12

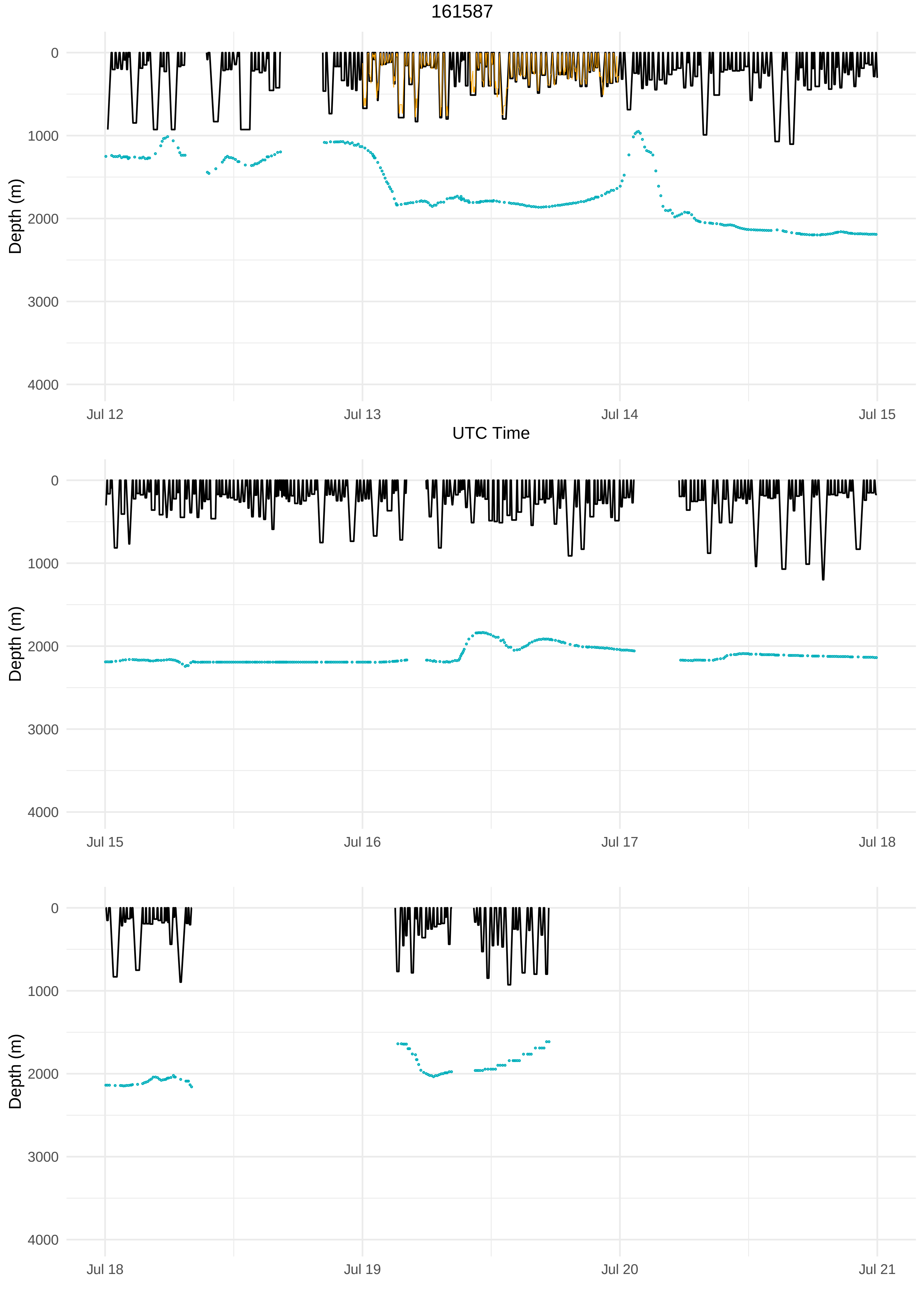

161587
0
1000
2000
3000
4000
Depth (m)
Jul 12
Jul 13
Jul 14
Jul 15
UTC Time
0
1000
2000
3000
4000
Depth (m)
Jul 15
Jul 16
Jul 17
Jul 18
0
1000
2000
3000
4000
Depth (m)
Jul 18
Jul 19
Jul 20
Jul 21

Figure S12.

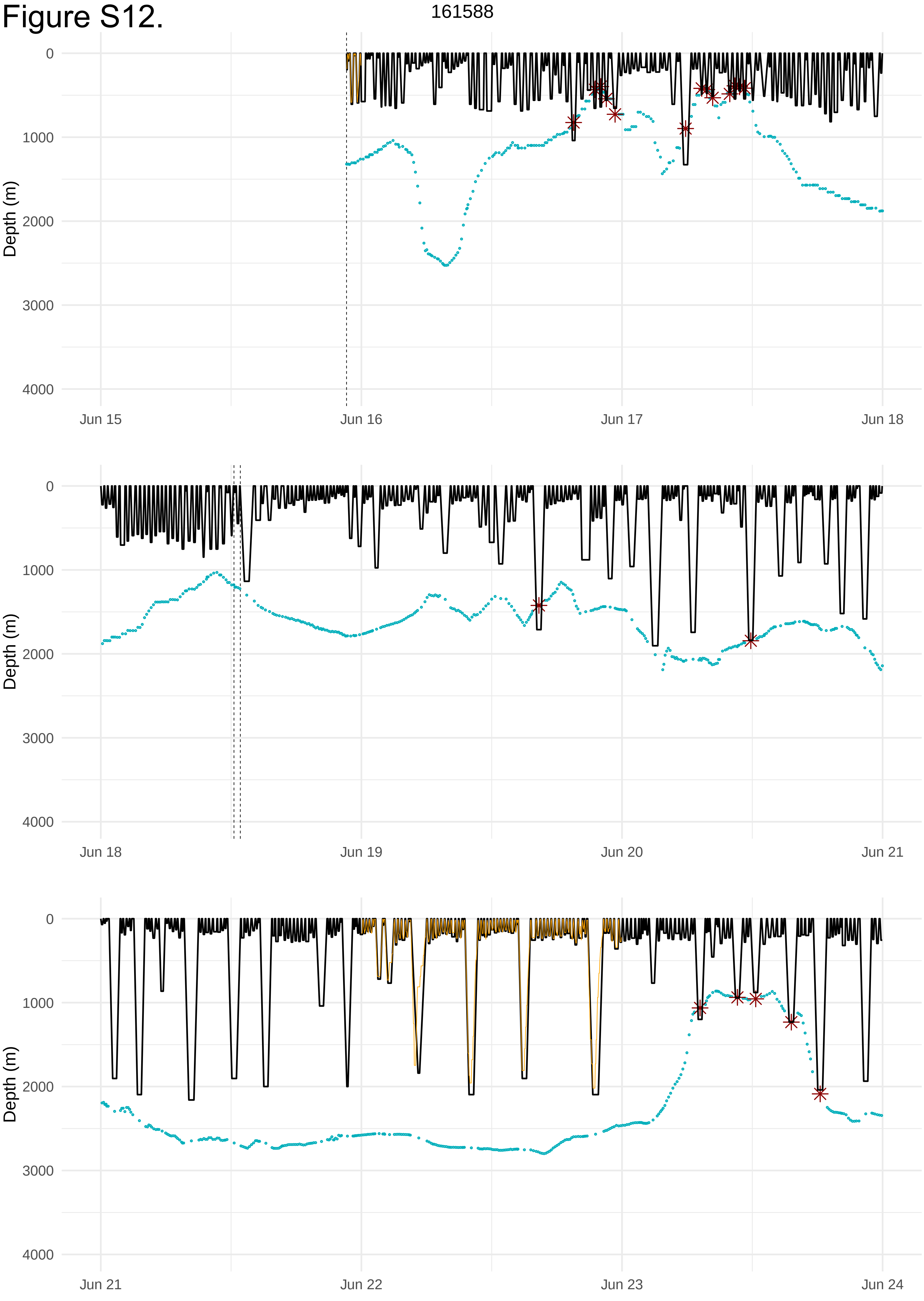

161588
0
1000
2000
3000
4000
Depth (m)
Jun 15
Jun 16
Jun 17
Jun 18
Jun 19
Jun 20
Jun 21
Jun 22
Jun 23
Jun 24

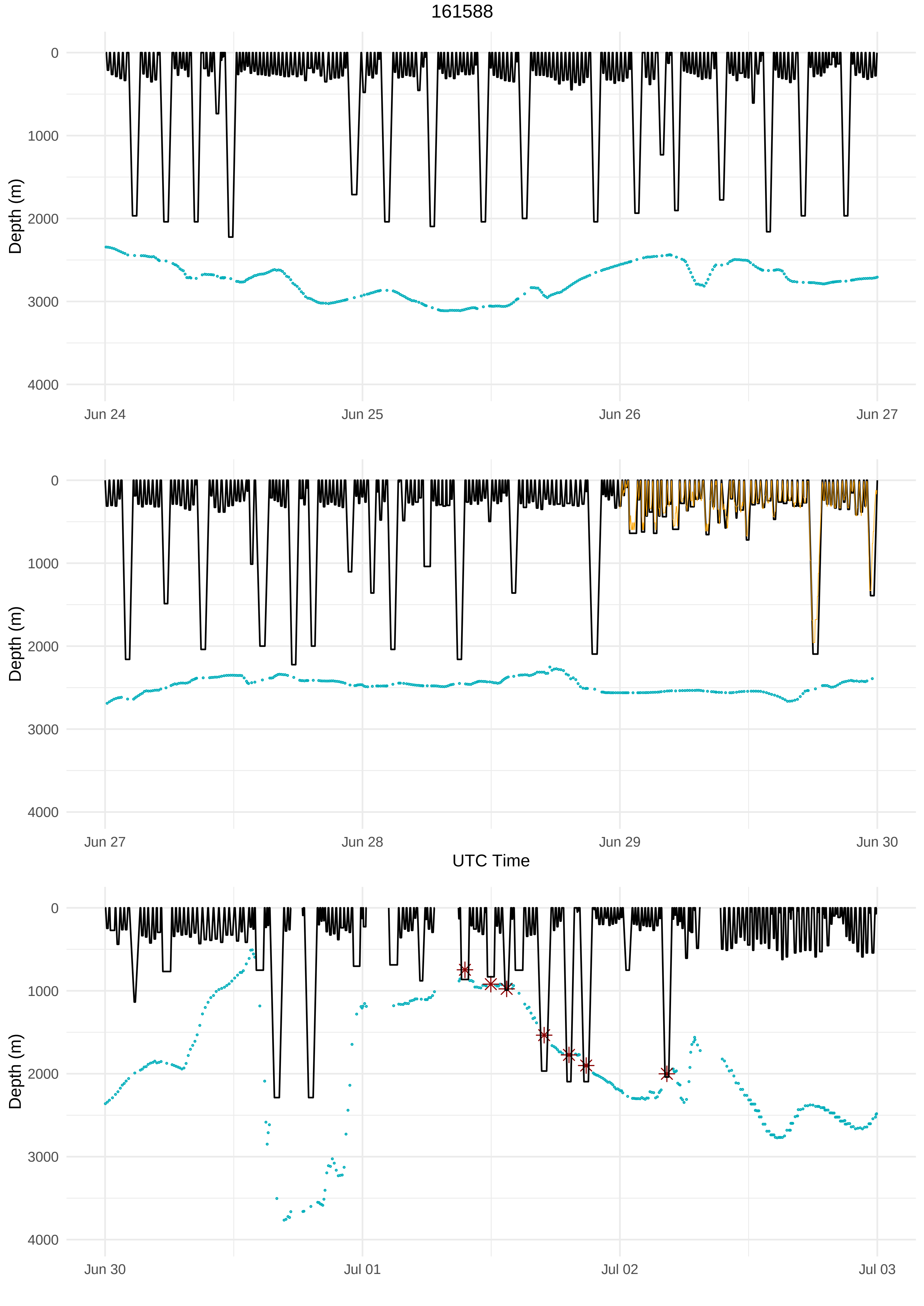
161588
Depth (m)
0
1000
2000
3000
4000
Jun 24
Jun 25
Jun 26
Jun 27
Jun 28
Jun 29
Jun 30
UTC Time
Jul 01
Jul 02
Jul 03

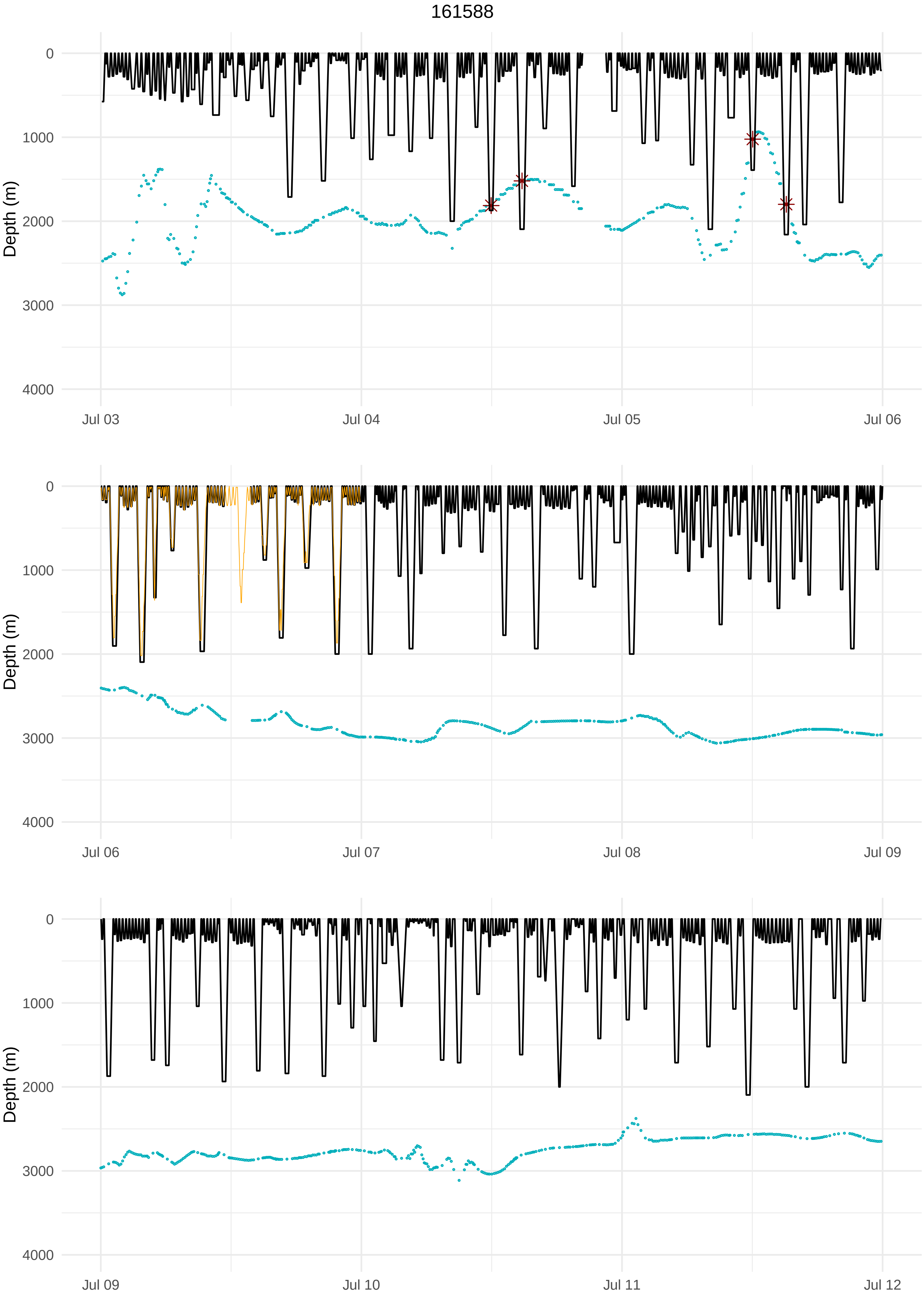

161588
Depth (m)
0
1000
2000
3000
4000
Jul 03
Jul 04
Jul 05
Jul 06
Jul 07
Jul 08
Jul 09
Jul 10
Jul 11
Jul 12

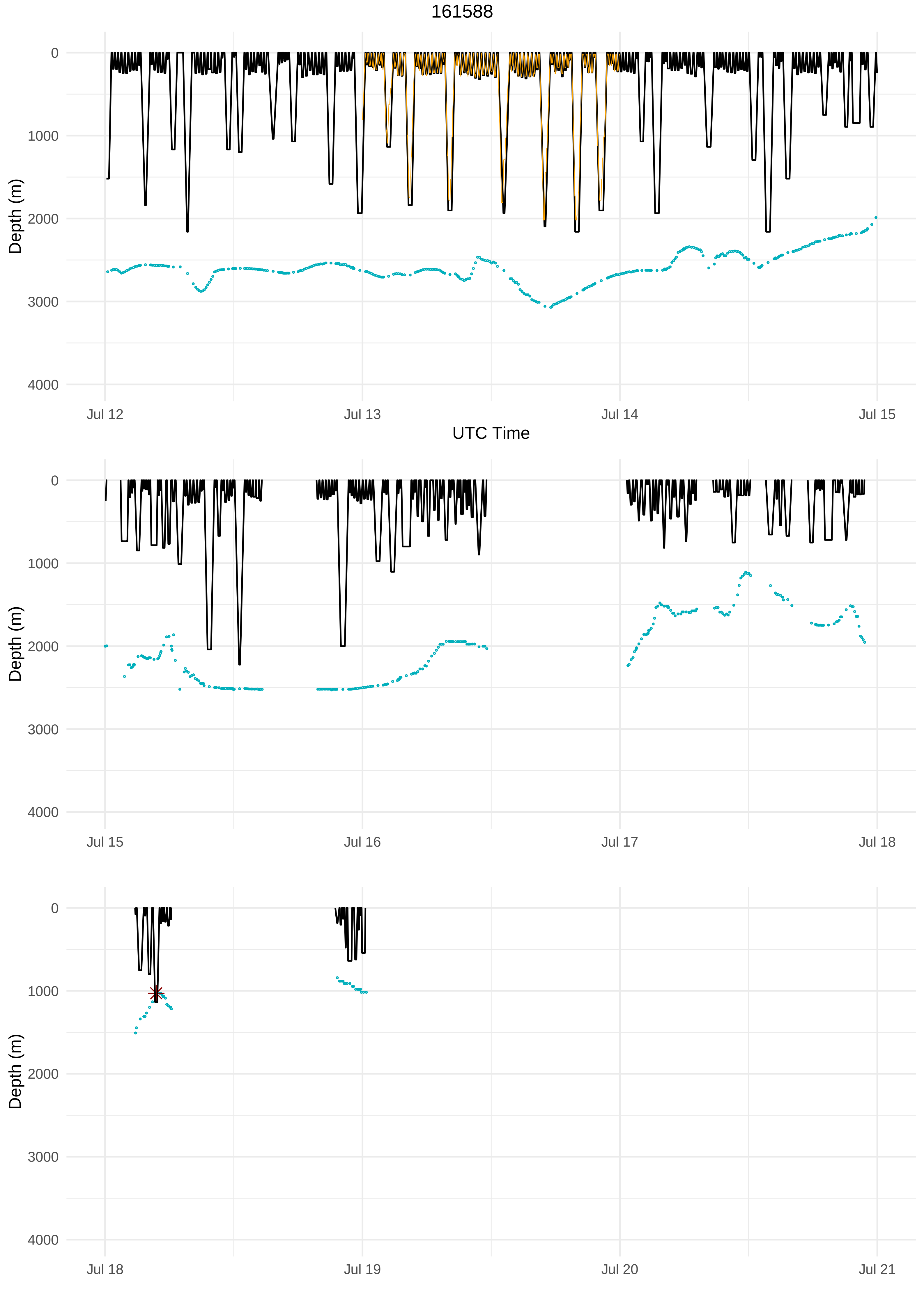

161588
Depth (m)
0
1000
2000
3000
4000
Jul 12
Jul 13
Jul 14
Jul 15
UTC Time
Jul 16
Jul 17
Jul 18
Jul 19
Jul 20
Jul 21

Figure S13.

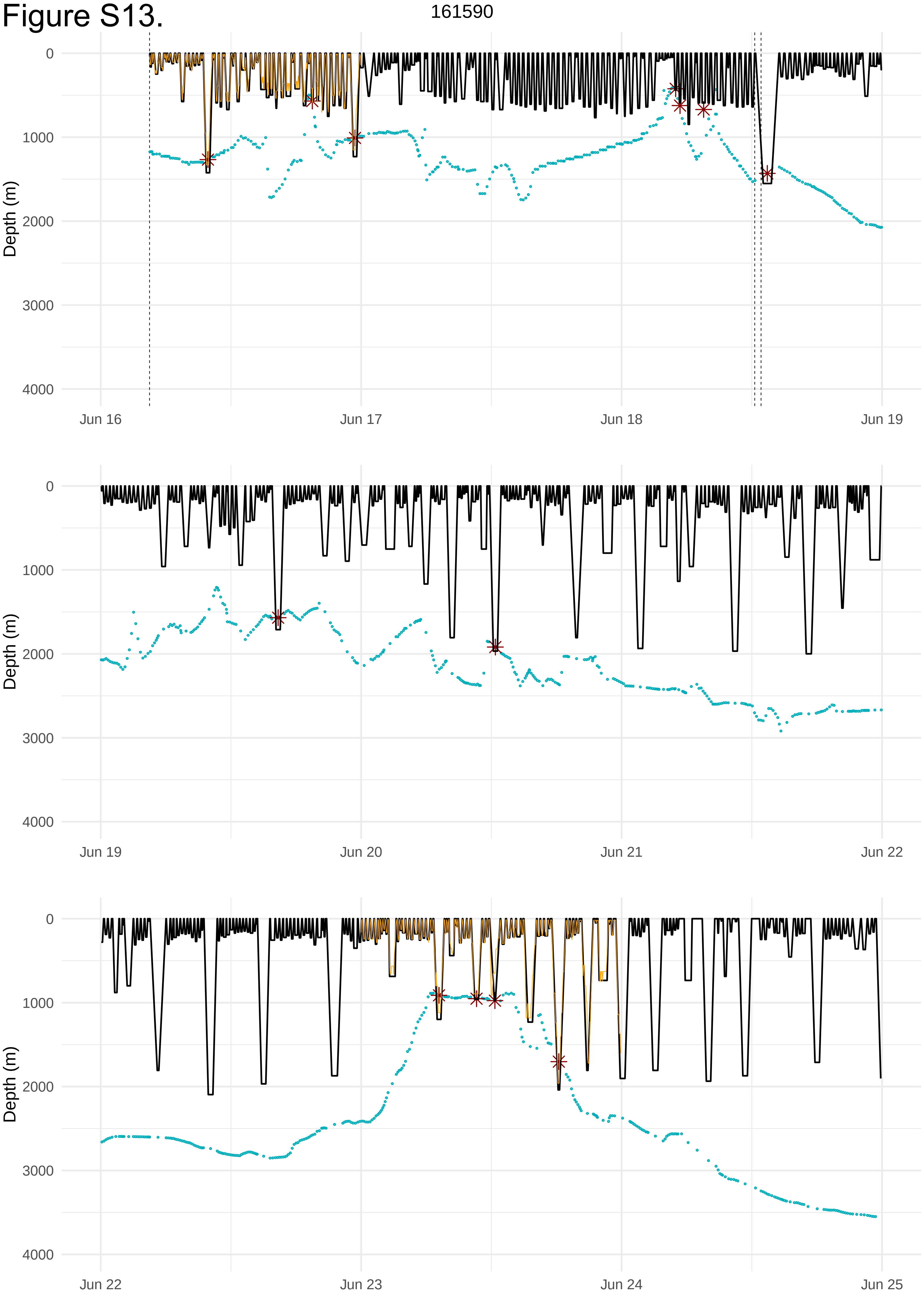

161590
Depth (m)
0
1000
2000
3000
4000
Jun 16
Jun 17
Jun 18
Jun 19
Jun 20
Jun 21
Jun 22
Jun 23
Jun 24
Jun 25

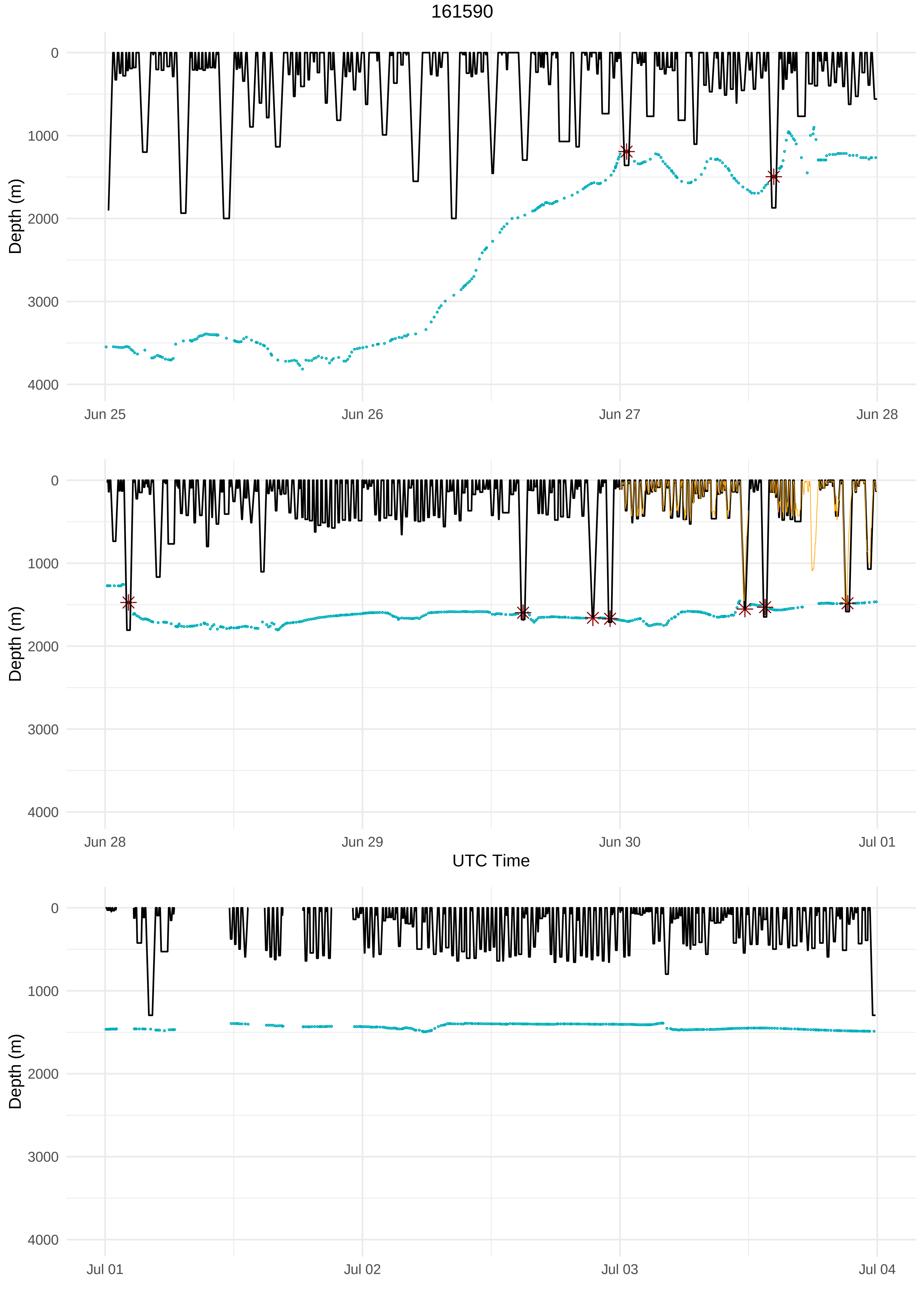

161590
Depth (m)
0
1000
2000
3000
4000
Jun 25
Jun 26
Jun 27
Jun 28
Jun 29
Jun 30
Jul 01
UTC Time
Jul 02
Jul 03
Jul 04

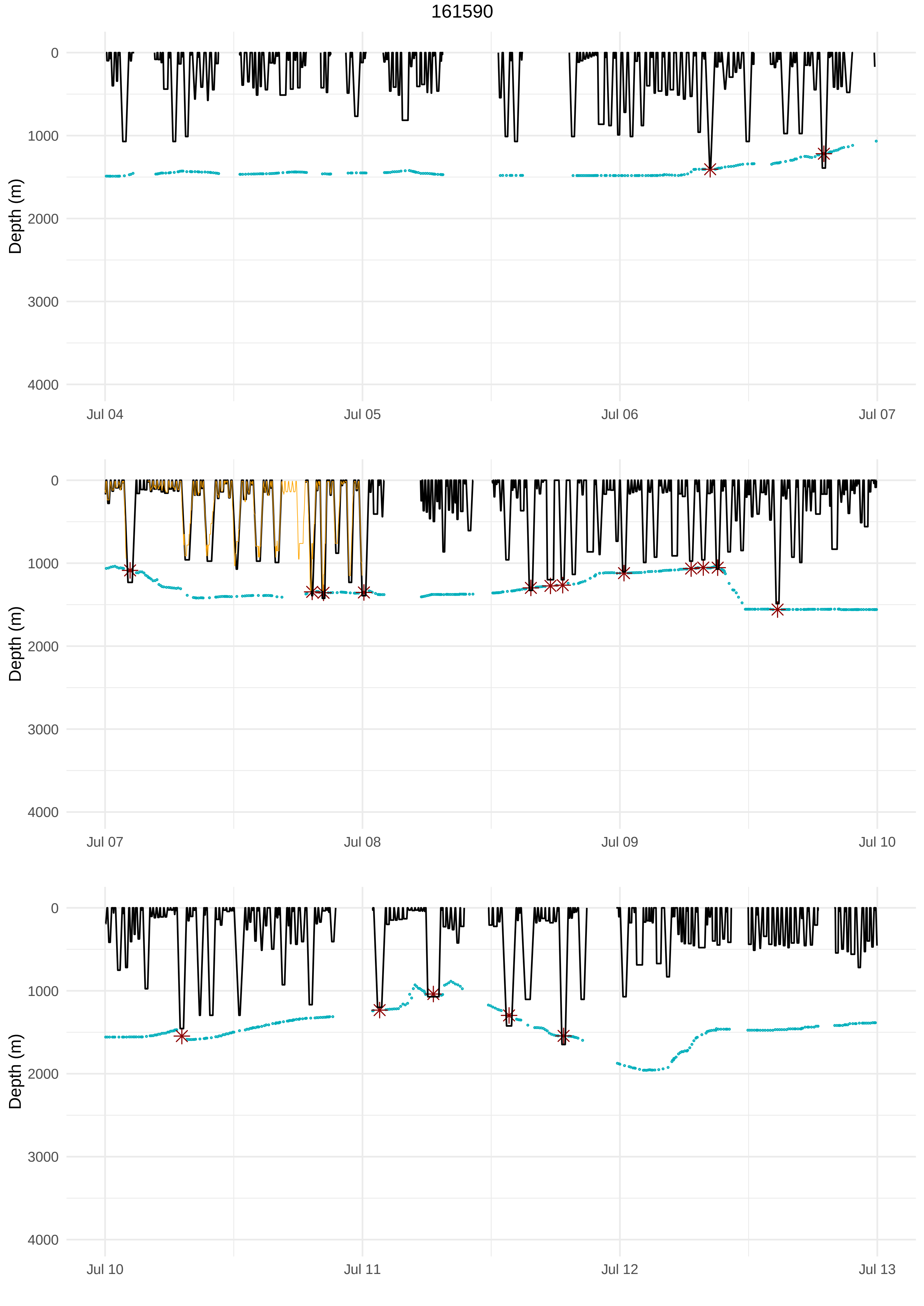
161590
Depth (m)
0
1000
2000
3000
4000
Jul 04
Jul 05
Jul 06
Jul 07
Depth (m)
0
1000
2000
3000
4000
Jul 07
Jul 08
Jul 09
Jul 10
Depth (m)
0
1000
2000
3000
4000
Jul 10
Jul 11
Jul 12
Jul 13

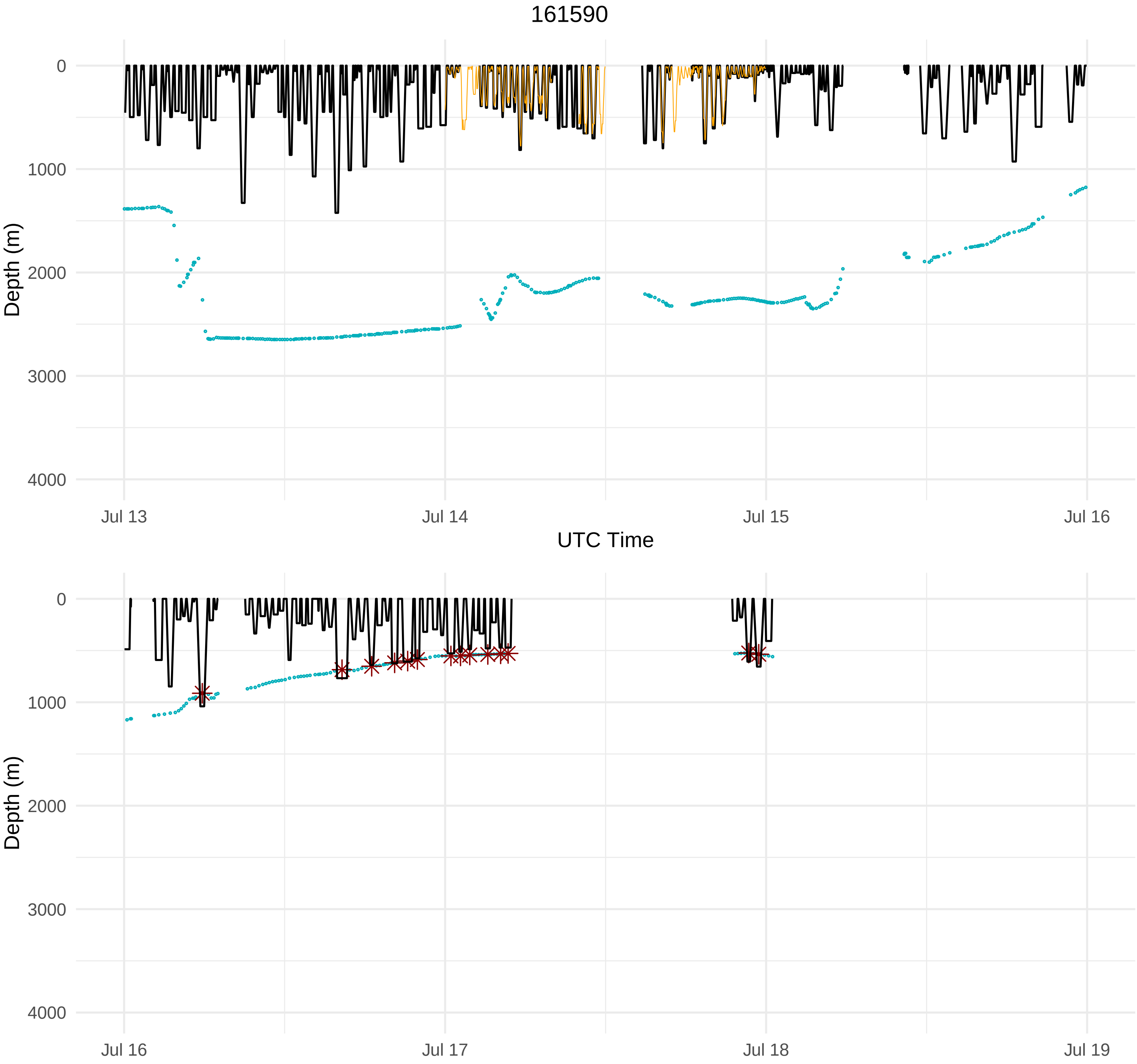
161590
0
1000
2000
3000
4000
Depth (m)
Jul 13
Jul 14
Jul 15
Jul 16
UTC Time
0
1000
2000
3000
4000
Depth (m)
Jul 16
Jul 17
Jul 18
Jul 19

Figure S14.

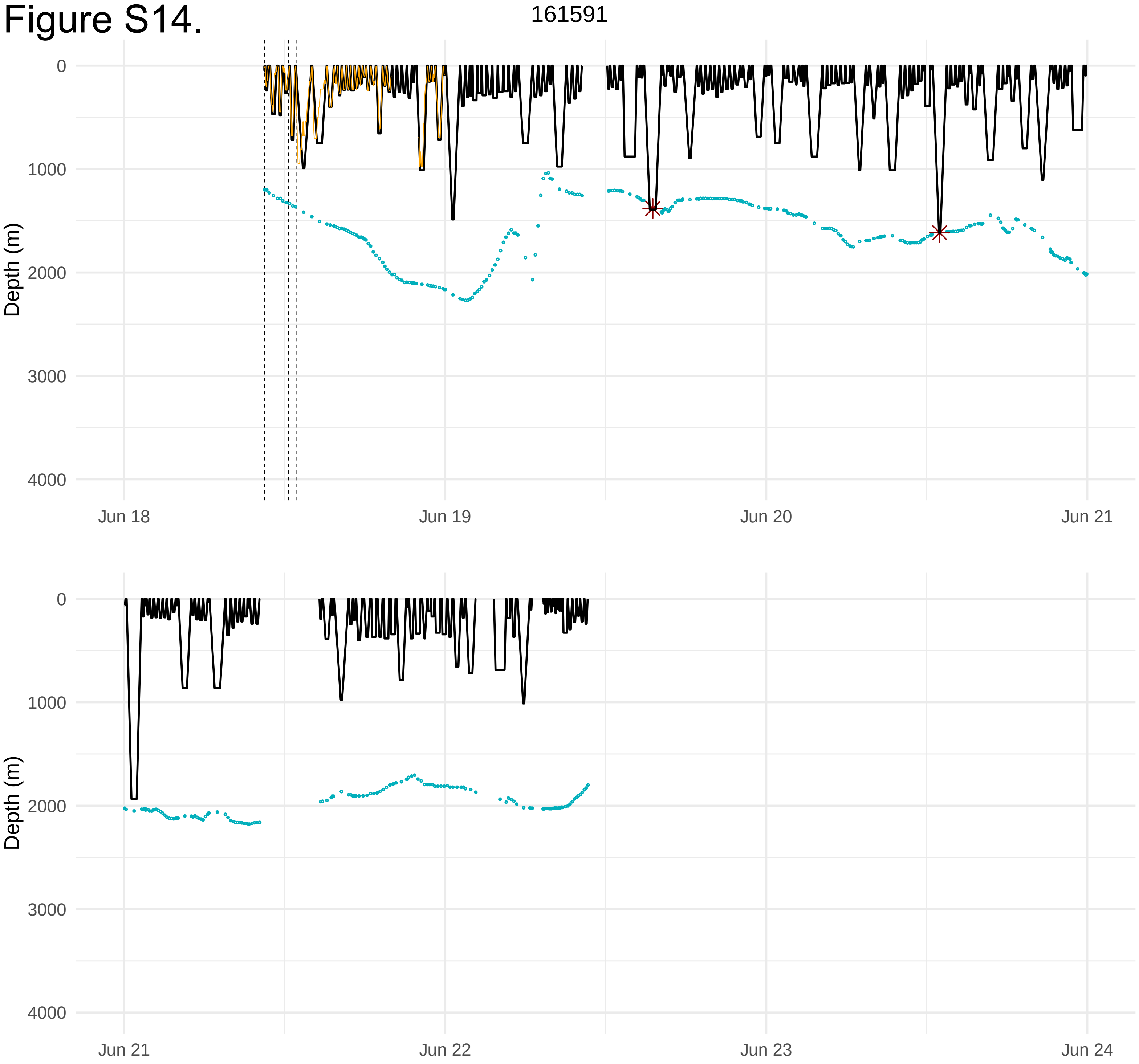

161591
Depth (m)
0
1000
2000
3000
4000
Jun 18
Jun 19
Jun 20
Jun 21
Jun 22
Jun 23
Jun 24

Figure S15.

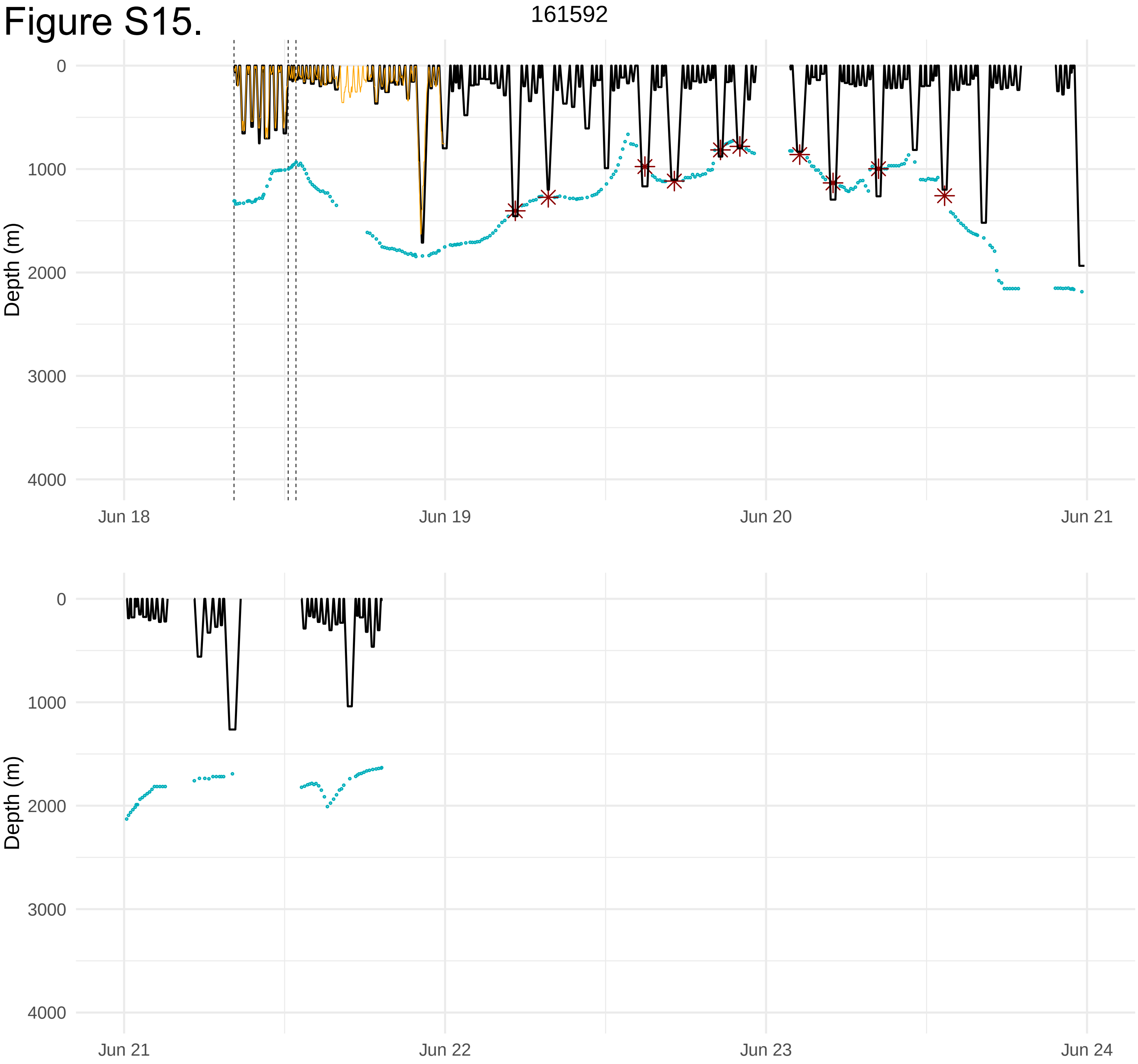
161592
Depth (m)
0
1000
2000
3000
4000
Jun 18
Jun 19
Jun 20
Jun 21
Depth (m)
0
1000
2000
3000
4000
Jun 21
Jun 22
Jun 23
Jun 24

Figure S16.

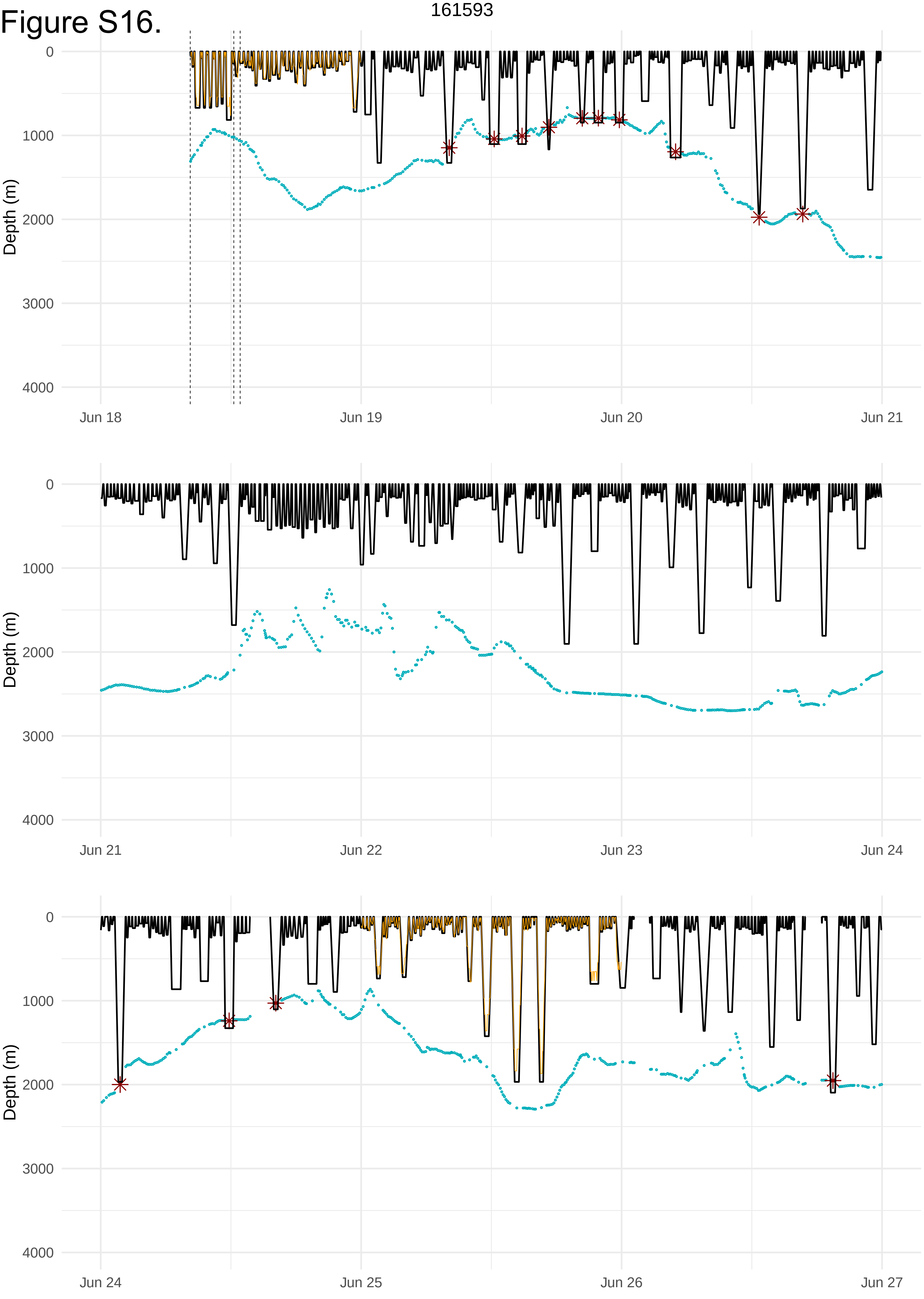
161593
Depth (m)
0
1000
2000
3000
4000
Jun 18
Jun 19
Jun 20
Jun 21
Depth (m)
0
1000
2000
3000
4000
Jun 21
Jun 22
Jun 23
Jun 24
Depth (m)
0
1000
2000
3000
4000
Jun 24
Jun 25
Jun 26
Jun 27

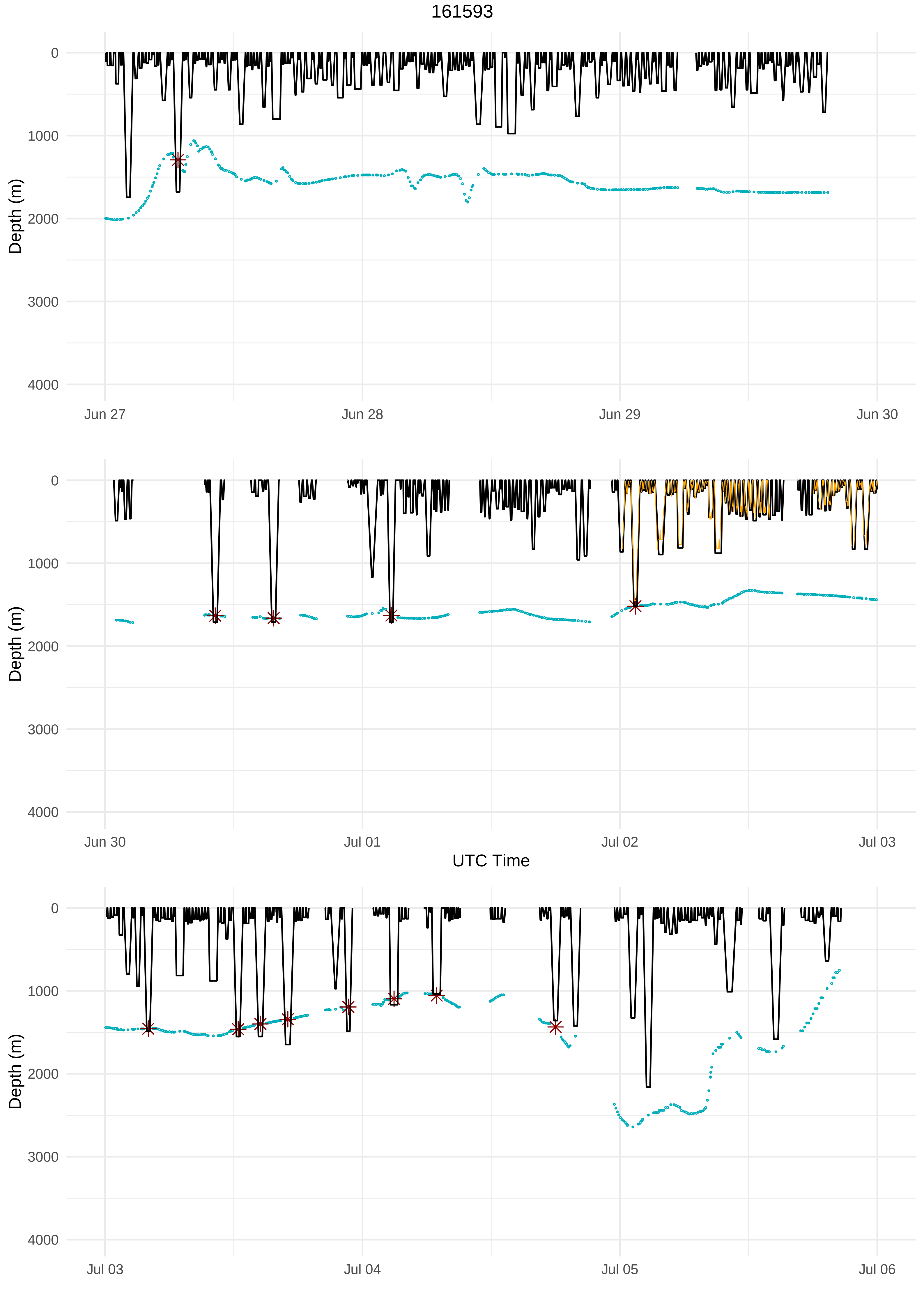

161593
Depth (m)
0
1000
2000
3000
4000
Jun 27
Jun 28
Jun 29
Jun 30
Jul 01
Jul 02
Jul 03
UTC Time
Jul 04
Jul 05
Jul 06

161593

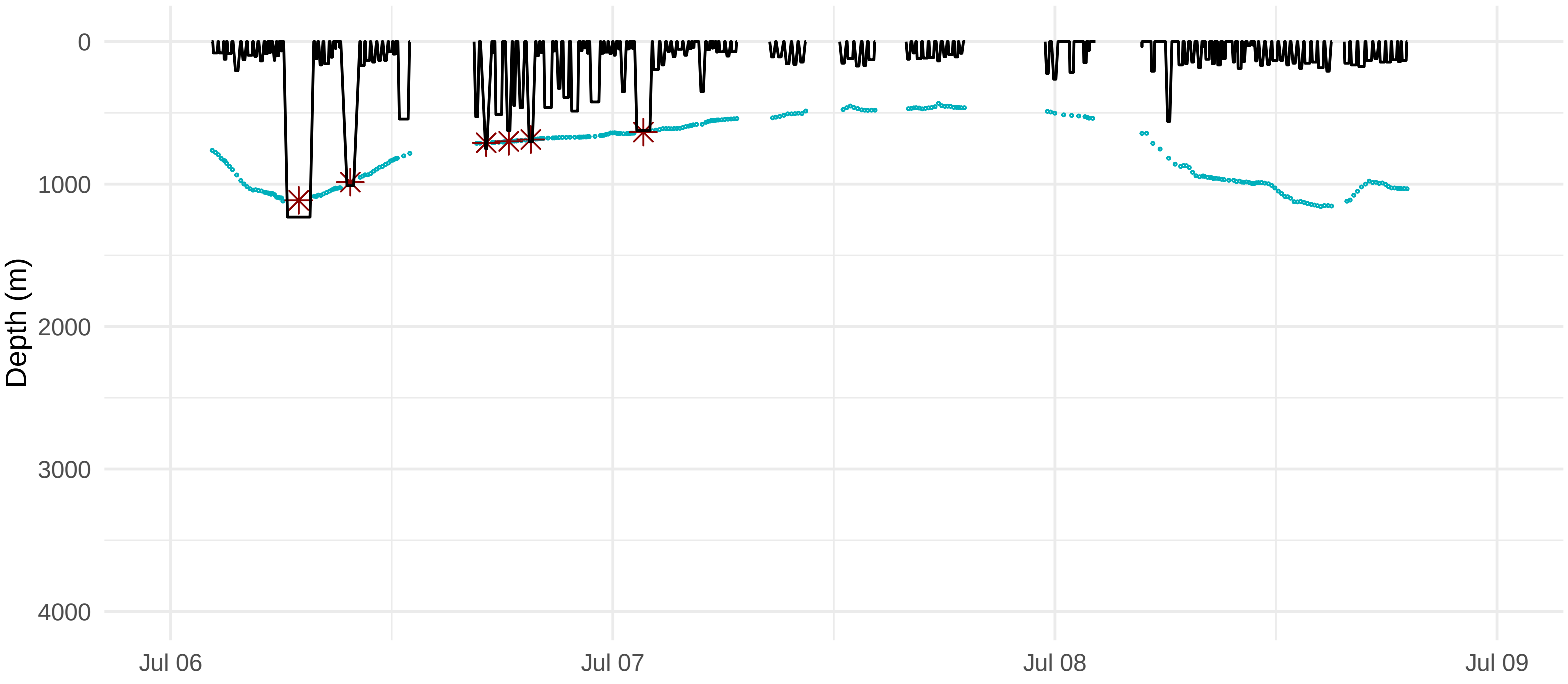

0
1000
2000
3000
4000
Depth (m)
Jul 06
Jul 07
Jul 08
Jul 09

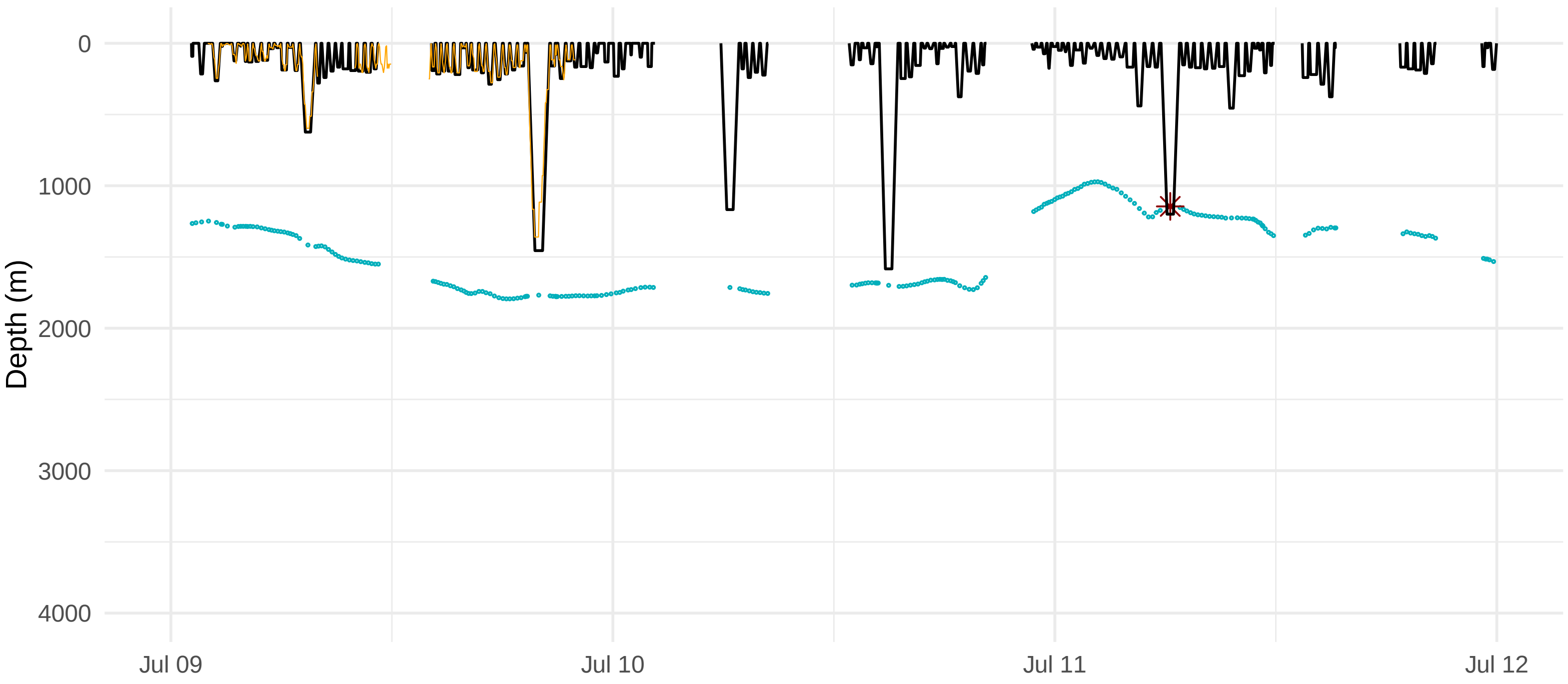

0
1000
2000
3000
4000
Depth (m)
Jul 09
Jul 10
Jul 11
Jul 12

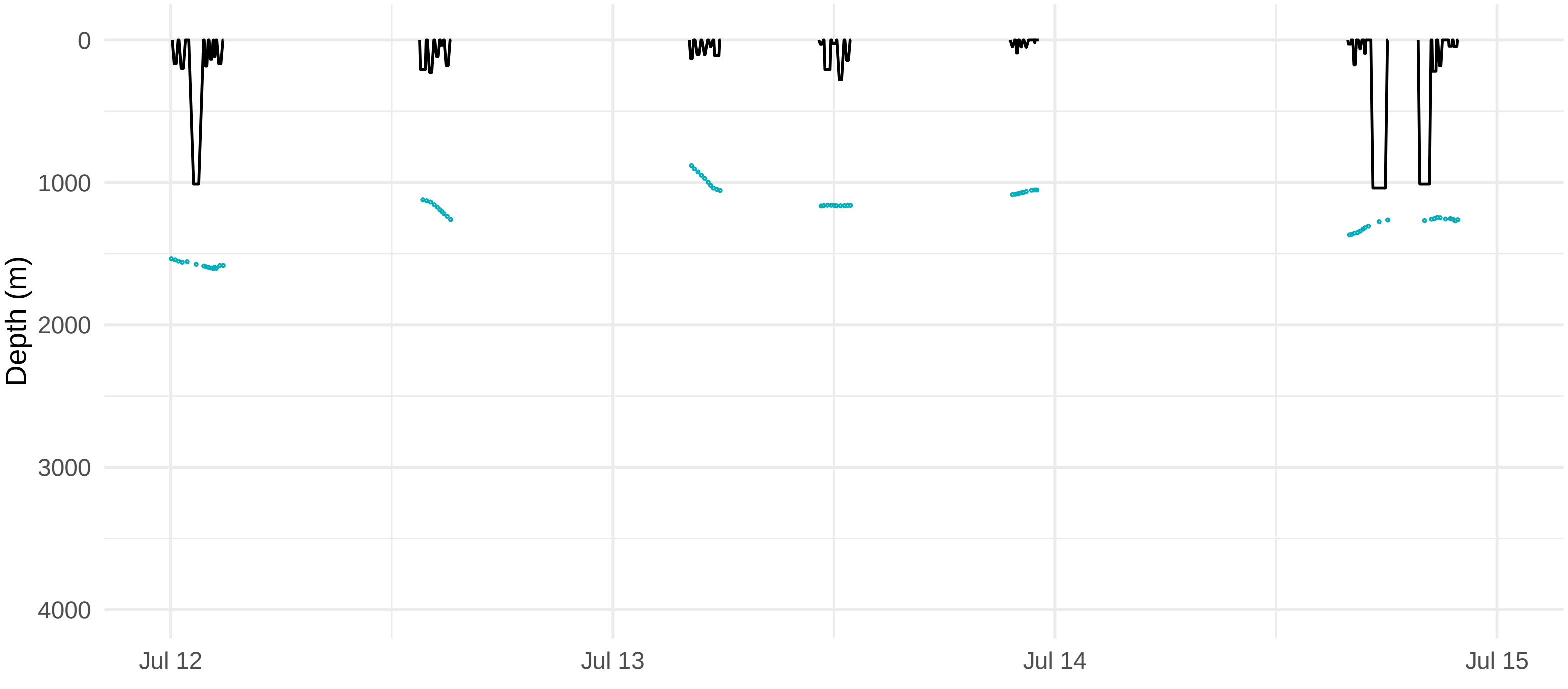

0
1000
2000
3000
4000
Depth (m)
Jul 12
Jul 13
Jul 14
Jul 15

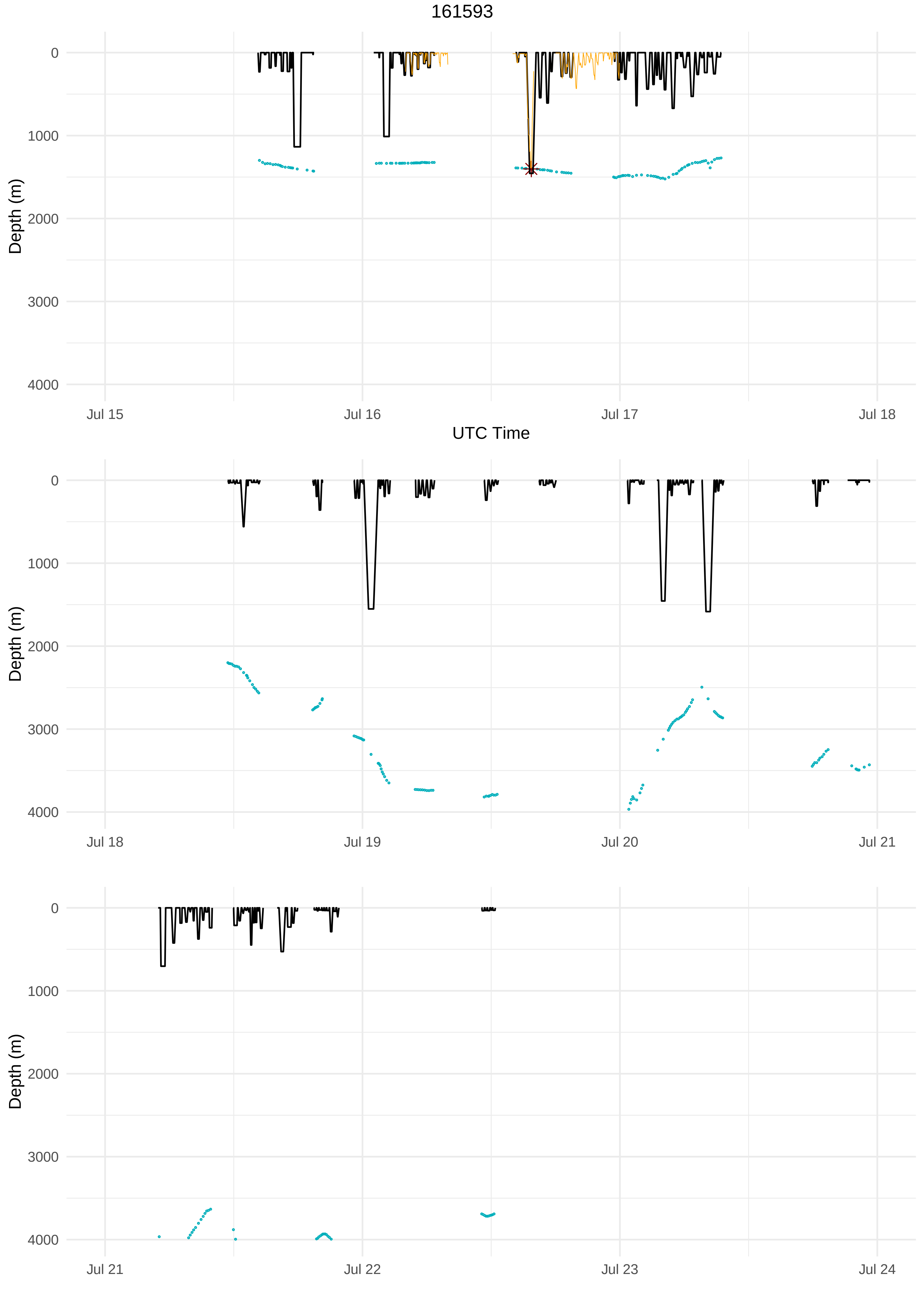

161593
0
1000
2000
3000
4000
Depth (m)
Jul 15
Jul 16
Jul 17
Jul 18
UTC Time
Jul 18
Jul 19
Jul 20
Jul 21
Jul 21
Jul 22
Jul 23
Jul 24

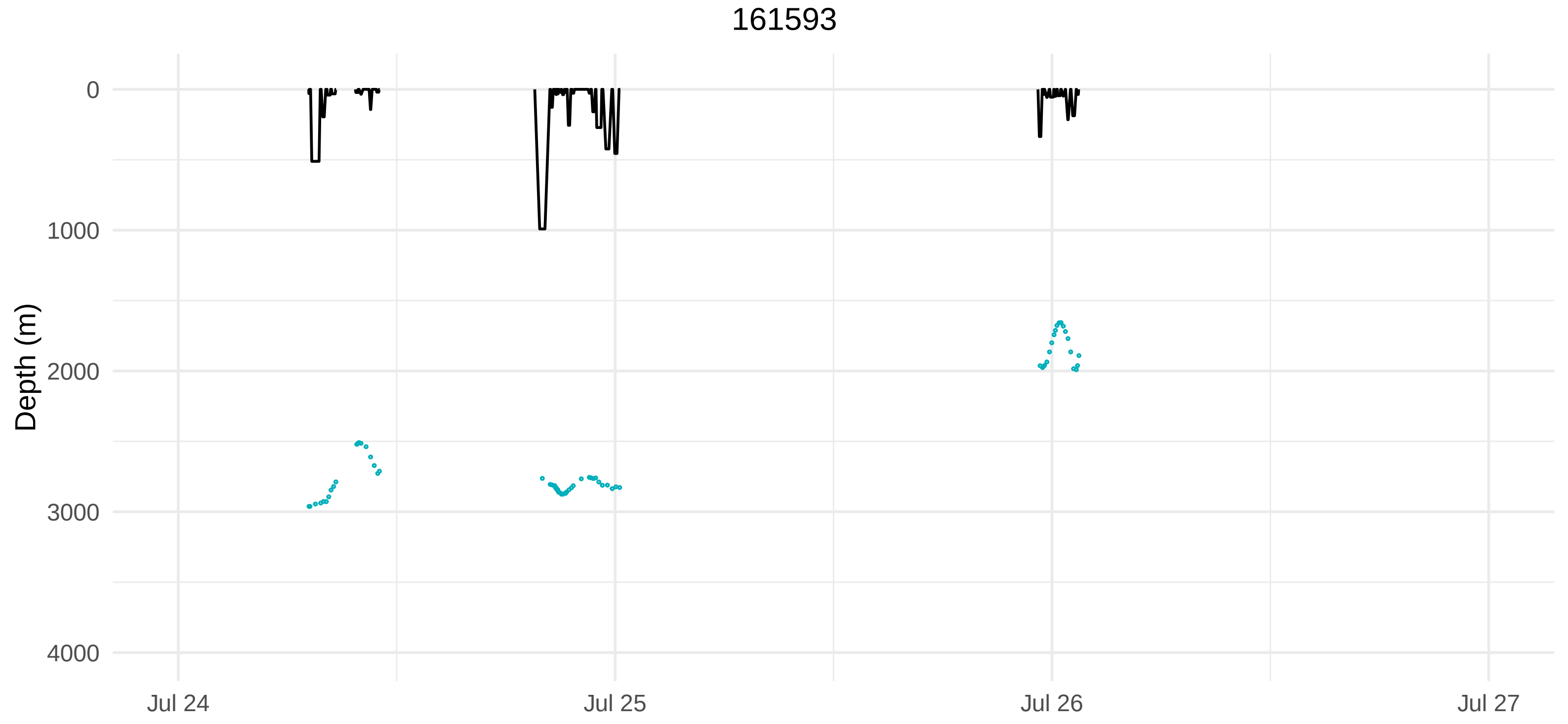
161593
Depth (m)
0
1000
2000
3000
4000
Jul 24
Jul 25
Jul 26
Jul 27
